# Bioactive Compounds from Citrus-Processing By-Products: A Narrative Review of Physical-Assisted and Conventional Extraction Technologies Plus Downstream Applications

**DOI:** 10.3390/molecules31173018

**Published:** 2026-08-28

**Authors:** Di Yang, Muze Yu, Yuanyuan Li, Lanlan Fang, Tingting Kuang, Jia Yu, Xiaoyan Tan, Jing Zhang, Ce Tang

**Affiliations:** 1 Laboratory for Innovation & Effective Uses of Chinese Drug Germplasm Resources, School of Ethnic Medicine, Chengdu University of Traditional Chinese Medicine, Chengdu 611137, China; yangdi@stu.cdutcm.edu.cn (D.Y.); yumuze@cdzyydx.wecom.work (M.Y.); liyuanyuan2@stu.cdutcm.edu.cn (Y.L.); fanglanlan@stu.cdzyydx.cn (L.F.); kuangtingting@stu.cdutcm.edu.cn (T.K.); yu77@cdutcm.edu.cn (J.Y.); 2 Chongqing Three Gorges Medical College, Chongqing 404120, China

**Keywords:** extraction methods, optimization, sustainability, bioactive compounds, pectin, essential oils

## Abstract

Citrus processing generates massive volumes of citrus-processing by-products rich in polyphenols, pectin, and essential oils, yet most of these materials are disposed of with low-value utilization, causing biomass loss and environmental pressure. Physical-assisted extraction techniques represent promising strategies for recovering high-value bioactive components from citrus-processing by-products. This narrative review assesses mainstream conventional and physical-assisted extraction routes (acid-assisted, ultrasound-assisted, microwave-assisted, and supercritical CO_2_ extraction) for phenolic compounds, pectin, and essential oils derived from citrus-processing by-products, summarizes optimal operational parameters, and compares their downstream food, biomaterial, and environmental remediation applications. Key findings demonstrate that ultrasound- and microwave-assisted extraction generally deliver higher extraction yields and better preservation of thermally sensitive bioactives relative to conventional reflux extraction; nevertheless, industrial-scale translation faces multiple bottlenecks, including high equipment investment, raw-material seasonal variability, incomplete solvent-recovery workflows, and scarce unified multi-dimensional evaluation benchmarks. Major limitations of existing research include the predominant focus on laboratory-scale yield optimization, with insufficient systematic quantitative comparisons covering energy consumption, product functional quality, life-cycle assessment (LCA), and techno-economic performance. Furthermore, thermal-driven oxidative degradation of d-limonene and polyphenols persists as a critical challenge hindering final product quality. Future perspectives highlight the need to establish standardized raw-material pretreatment protocols, combine chemical-based evaluation metrics and ISO 14040-compliant LCA frameworks to balance environmental benefits and economic profitability, and advance pilot-scale validation for hybrid coupled extraction processes. Key conclusions: although citrus-processing by-products possess enormous biorefinery potential, bridging laboratory-scale feasibility and industrial commercialization still requires joint progress in extraction-process optimization, safety validation, and circular-economy-oriented technical innovation.

## 1. Introduction

Citrus fruits, belonging to the Rutaceae, are economically valuable crops widely cultivated throughout tropical and subtropical regions of the world. Their fruits are highly favored by consumers for their bright color, rich aroma, and distinctive flavor [1]. According to the FAOSTAT database [2], global citrus fruit production reached approximately 143 million tonnes in 2022, with China as one of the dominant citrus producers, contributing roughly 40% of the world’s total output. About 14% of citrus fruits are processed into products such as juice, wine, jam, candies, and seasonings, generating large amounts of by-products in the process, including peels, seeds, pomace, and residual pulp. Among them, the peel accounts for approximately 50% of the total fruit weight and is rich in pectin, phenolic compounds, dietary fiber, essential oils, and other bioactive components, thus possessing significant potential for reuse [3,4]. Research into the current industry indicates that the overall resource utilisation rate of by-products from citrus processing worldwide remains below 40 per cent; large quantities of citrus-processing by-products are disposed of at low value or even discarded outright. A targeted narrative overview summarising citrus-processing by-products valorization technologies across the whole value chain possesses both theoretical and practical significance to advance the citrus circular economy system [2,3,5].

The utilization of agricultural and industrial by-products has become a key strategy for improving resource use efficiency. This approach recovers valuable components that would otherwise be discarded, thus helping to reduce environmental burdens. Recent studies have widely explored the integrated use of peels from fruits such as banana [6], watermelon [7], apple, and grape [8]. citrus-processing by-products has attracted particular attention due to its richness in pectin, polyphenols, pigments, and polysaccharides. These components demonstrate significant application potential in areas such as microencapsulation [9], food additives [10], biodegradable films [11], and adsorbent materials for water treatment [12].

However, the extraction efficiency of target compounds from citrus-processing by-products is affected by various factors, including variety, ripeness, and the extraction method. Currently, no unified and efficient extraction standard exists. Therefore, optimizing extraction methods is crucial for improving recovery rates, reducing energy consumption, and lowering production costs [13]. Moreover, the chemical composition of peels varies significantly across different varieties, necessitating the development of suitable extraction and conversion strategies for each target compound [5].

A wide range of extraction technologies have been developed to recover bioactive compounds from citrus-processing by-products, including ultrasound-assisted extraction (UAE), microwave-assisted extraction (MAE), high-pressure processing (HPP), pulsed electric field (PEF) and supercritical fluid extraction (SFE). Among them, UAE and MAE are the most widely investigated methods, owing to their high extraction efficiency, low solvent consumption and favorable potential for industrial scale-up [14,15]. Meanwhile, conventional acid extraction remains the dominant industrial technique for commercial pectin production [16]. HPP, PEF and SFE are limited by expensive equipment and insufficient industrial verification [15,17]. Therefore, this review focuses on conventional acid extraction, UAE and MAE. We systematically compare these techniques in terms of extraction efficiency, as well as the physicochemical, biological and functional properties of the recovered pectin, polyphenols and essential oils. The respective strengths and limitations of each extraction approach are comprehensively discussed.

Multiple review papers have previously summarized citrus-processing by-products biorefinery and bioactive component recovery. Most existing reviews either focus on a single class of active substances, discuss only one extraction technology, or merely describe a single application scenario. Few publications systematically compare acid, ultrasound-assisted and microwave-assisted extraction for simultaneous recovery of pectin, polyphenols and essential oils from diverse citrus varieties. Furthermore, limited literature contrasts the functional properties of extracts obtained by these three mainstream techniques and links extraction performance with downstream application prospects. In addition, summaries concerning industrialization bottlenecks are often fragmented [18]. Against such a research background, it is necessary to construct a focused comparative analysis to fill these knowledge gaps.

To ensure focused and comprehensive literature screening, we conducted a targeted literature search in Web of Science, Scopus, and PubMed using core keywords related to citrus-processing by-products extraction technologies and bioactive components, including “citrus peel extraction”, “acid extraction”, “ultrasound-assisted extraction”, “microwave-assisted extraction”, “pectin”, “polyphenols”, and “essential oils”. To fully cover the downstream high-value utilization content of this review, additional application-oriented keywords were also adopted, such as “citrus peel application”, “food fortification”, “biodegradable film”, “antioxidant”, “antimicrobial”, and “environmental remediation”. Studies published between 2015 and 2026 were considered, with priority given to literature reporting quantitative extraction parameters, compound characterization data, and practical application performance. Given the narrative nature of this review, we did not apply strict inclusion/exclusion criteria or formal quality assessment procedures. Instead, we selected representative studies that enable meaningful cross-technology comparisons across different citrus varieties and target compounds, as well as comprehensive analysis of their diversified application prospects.

Despite the growing body of review publications addressing citrus-processing by-products valorization, most existing reviews tend to emphasize extraction-yield performance while paying insufficient attention to the functional quality of recovered bioactive constituents. Many previous summaries only offer separate descriptions of individual extraction technologies without performing structured cross-technique comparisons across multiple practical dimensions, such as energy input, structural preservation of target compounds, industrial scalability, life-cycle environmental impacts and techno-economic feasibility. In addition, few reviews integrate green-chemistry evaluation criteria and ISO 14040-aligned life-cycle assessment evidence to identify real-world bottlenecks restricting industrial translation. Gaps also remain concerning systematic collation of compound stability risks, including thermal-oxidative degradation of limonene and polyphenols during processing [19]. Against this background, the present narrative review focuses on physical-assisted extraction pathways. It aims to compare mainstream extraction approaches not merely based on extraction yields but also from the perspective of product functional characteristics, analyze key limiting factors for scale-up, summarize multi-field downstream applications, and highlight priority directions for future research to support the circular-economy-oriented biorefinery of citrus-processing by-products.

This work presents a narrative review focusing on conventional and physical-assisted extraction methods for key functional components isolated from various citrus-processing by-products. Its novelty lies in the targeted comparative evaluation of three industrially viable extraction routes (acid extraction, UAE, and MAE) for the simultaneous recovery of multiple bioactive compounds. We compile optimal processing parameters of different extraction techniques and selectively evaluate recent advances in food, materials, and environmental applications of citrus-processing by-products-derived functional products. This review aims to provide theoretical support and technical references for the efficient, sustainable, and high-value valorization of citrus-processing by-products.

## 2. Structure, Chemical Components and Medicinal Value of Citrus

**Figure 1:**
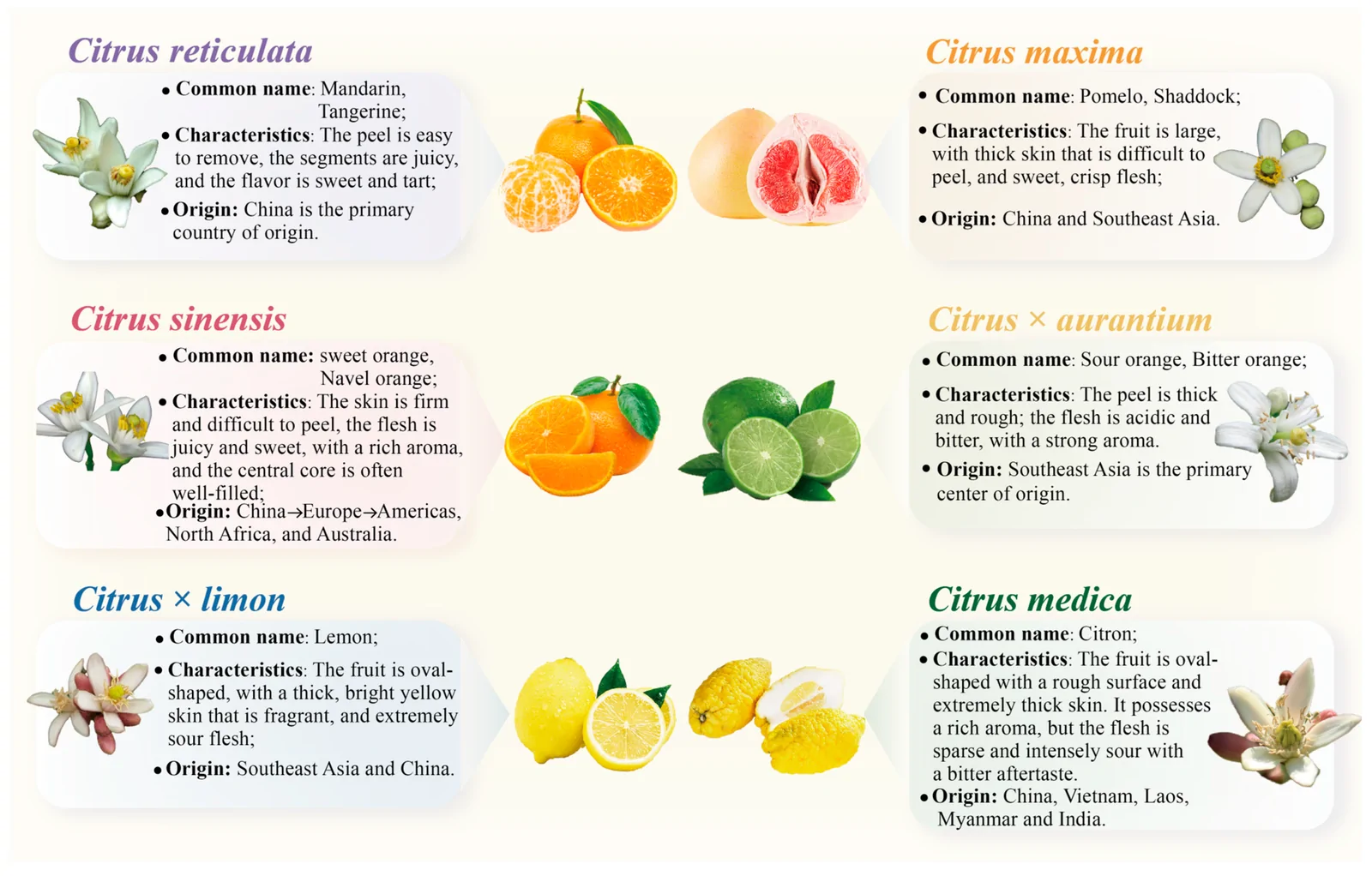
Major differences among common citrus varieties.

The average weight of citrus fruits differs across varieties, as shown in Figure 1. The fruit’s structure primarily consists of edible pulp, which accounts for 50–70% of the total weight. The peel makes up approximately 30–50%, with seeds comprising a small fraction [20]. Citrus fruits display notable sensory characteristics, including rich hues ranging from orange to yellow and green, juicy pulp consisting of plump sacs, and textured rinds, which are well-suited for essential oil extraction [21]. During flowering, citrus blossoms display vibrant colors, with petal lengths varying according to cultivar and fruit hue.

In traditional Chinese medicine and in contemporary biomedical research, citrus peels from different species and maturity stages are recognized to exhibit distinct medicinal properties. In particular, the unripe peel of *Citrus reticulata* Blanco, known as Qingpi, is commonly used in crude or vinegar-processed form in traditional formulations. It is rich in polymethoxyflavones, such as hesperidin and nobiletin. In vitro chemical assays and cell-based studies have documented notable anti-inflammatory and antioxidant activities, while animal experiments have suggested regulatory effects on gastrointestinal motility [16,22]. However, these preclinical findings remain preliminary; their relevance to human health is uncertain, as the biological effects of these compounds are largely constrained by low bioaccessibility and poor bioavailability after oral administration, and no well-controlled human studies have yet confirmed their clinical efficacy. In contrast, the mature peel of citrus fruits, namely Chenpi (e.g., Xinhui Chenpi), undergoes drying and aging, during which its volatile oil composition changes substantially. The essential oil, with limonene as the predominant component, acts synergistically with chenpi glycosides and is widely used for regulating qi, strengthening the spleen, and resolving dampness and phlegm. Modern preclinical animal studies further indicate its potential to modulate gut microbiota and lipid metabolism [23,24]. Nevertheless, as with Qingpi, these observations are derived from animal models and require further validation through pharmacokinetic and clinical investigations. In addition, the dried exocarp of sweet orange (*Citrus sinensis*) is also frequently used as a medicinal excipient or as a source for flavonoid extraction, and its pharmacological activities are closely associated with the processing conditions [25]. It should also be noted that excessive intake of concentrated citrus peel extracts may raise toxicological concerns; comprehensive safety evaluation is required before supporting any systemic health claims.

Citrus fruits are widely consumed for their appealing flavor and nutritional value. Both the pulp and peel contain abundant bioactive compounds, including vitamin C, fiber, minerals, polyphenols, and essential oils, among which antioxidant constituents are particularly prominent [26]. Although the peel is usually regarded as a processing by-product, it constitutes a valuable source of nutrients and functional ingredients with considerable industrial and health-related application potential. [27]. The scientific community is currently exploring efficient methods for extracting high-value components from citrus peel to enhance its comprehensive utilization potential.

## 3. Extraction of Compounds Present in Citrus Peels

**Figure 2:**
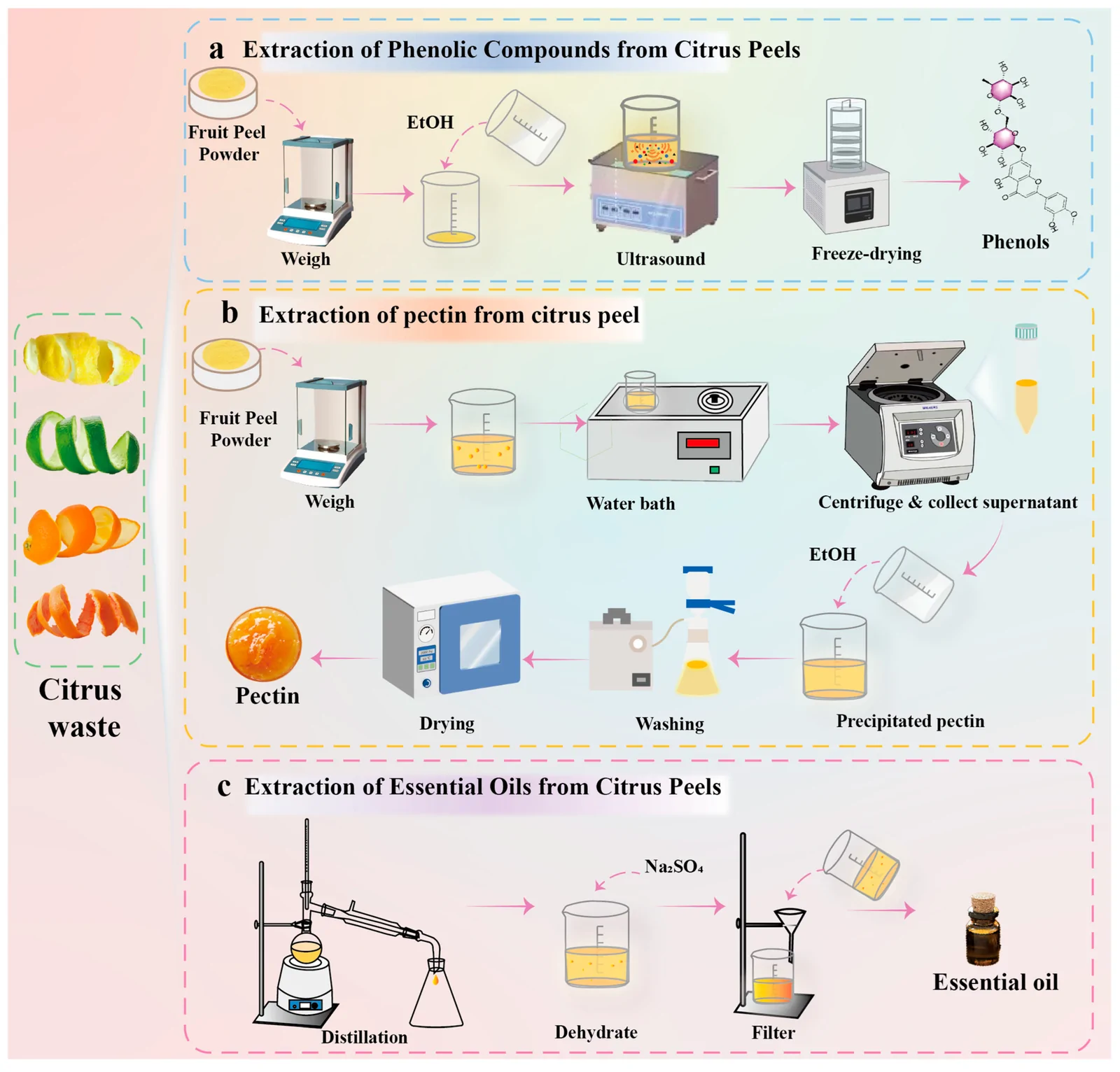
Extraction workflows of bioactive components from citrus peels. (**a**) Ultrasound-assisted extraction of phenolic compounds with subsequent freeze-drying; (**b**) Isolation procedure of pectin, covering water-bath extraction, centrifugation, ethanol precipitation, washing and drying; (**c**) Distillation-based extraction process of essential oils from citrus peels, involving dehydration with anhydrous sodium sulfate and filtration to collect final essential-oil products.

Citrus peel contains various compounds with potential industrial applications, such as phenolic compounds, pectin, and essential oils. Optimizing extraction methods (Figure 2) can significantly enhance extraction efficiency and product quality while reducing production costs and resource consumption, thus achieving a more environmentally friendly and economically efficient production process.

### 3.1. Bioactive Compounds

The content of polyphenols, pectin and essential oils in citrus peel is influenced by multiple factors, including variety, growing region, ripeness, field cultivation practices, and post-harvest storage and transport. Significant fluctuations in the composition of the raw material can directly alter the optimal extraction process parameters; however, existing studies predominantly utilise a single raw material, lacking parallel comparisons across multiple origins and varieties, making it difficult to establish a universal, standardised extraction protocol [28].

Bioactive compounds are naturally occurring substances found in foods and plants. In chemical assays and in vitro cell models, many bioactive compounds exhibit antioxidant capacity to suppress lipid peroxidation, and preclinical animal studies suggest a broad spectrum of bioactivities, including potential neuroprotective, anticancer, anti-inflammatory, cardioprotective and antidiabetic effects. Nevertheless, such in vitro and animal-derived observations cannot be directly extrapolated to clinical efficacy in humans. After oral intake, the biological actions of these compounds are strongly limited by low bioaccessibility and bioavailability [29]. As major sources of natural phytochemicals, fruits and vegetables have been widely investigated for health-promoting effects. Epidemiological studies indicate an inverse correlation between higher fruit and vegetable consumption and the incidence of multiple chronic diseases, although direct causal relationships cannot be fully confirmed based on current evidence [30].

Citrus processing generates substantial processing by-products, including peels, seeds and pomace. These materials are rich in bioactive compounds, including pectin, essential oils, as well as hydrophilic and lipophilic antioxidants [31]. For example, citrus peel residue, a byproduct of the juice industry, contains abundant pectin, dietary fiber, phenolic compounds, essential oils, and other antioxidant-active components [26]. Similarly, grape pomace contains anthocyanins [32] (He et al., 2023), carrot pulp is rich in carotenoids [33], and phenolic compounds are extractable from dragon fruit peel [34].

Phenolic compounds are among the most abundant secondary metabolites in citrus fruits and are mainly classified into phenolic acids and flavonoids. Flavonoids further include subclasses such as flavanones, flavonols, and polymethoxyflavones, which are mostly present in free or glycosylated forms [35]. In laboratory investigations, these metabolites can interact with gut microbiota and the gastrointestinal milieu, retain structural integrity against oxidative stress and alleviate oxidative damage [36]. Citrus fruits also accumulate carotenoids, which are responsible for orange and yellow pigmentation and display strong antioxidant activity in in vitro tests [37]. Flavonoids bear phenolic hydroxyl groups, conferring amphipathic and weakly acidic properties. In in vitro and preclinical settings, flavonoids demonstrate favourable antioxidant, anti-inflammatory and immunomodulatory properties. Even so, further controlled human trials are required to validate their practical value for supporting human health and chronic disease risk management. Phenolic and terpenoid compounds are the most extensively studied bioactive components in citrus fruits, found in both the peel and pulp. Studies show that the concentration of bioactive compounds in citrus peel typically exceeds that in the pulp [38]. The content of these compounds varies significantly depending on factors such as citrus variety, origin, and ripeness, while extraction methods notably affect both yield and structural integrity [39,40].

During extraction, phenolic stability is highly dependent on processing conditions, including temperature, extraction duration, solvent pH, solvent polarity, and oxygen exposure. High temperature, prolonged treatment, and alkaline microenvironments accelerate phenolic degradation through multiple chemical pathways, including quinone transformation, glycosidic bond hydrolysis, and aromatic ring-opening oxidation. These degradation mechanisms alter phenolic monomer profiles, reduce total phenolic retention, and substantially weaken antioxidant bioactivity[41]. In contrast, mild, short-duration physical-assisted extraction can effectively suppress thermal-oxidative deterioration and preserve native phenolic structures.

For analytical characterisation, total phenolic content is routinely quantified via the Folin-Ciocalteu spectrophotometric method [42]. Individual phenolic monomers, including hesperidin, naringin, and phenolic acids, are qualitatively identified and quantitatively determined using high-performance liquid chromatography with diode-array detection (HPLC-DAD) and ultra-performance liquid chromatography coupled with quadrupole time-of-flight tandem mass spectrometry (UPLC-Q-TOF-MS/MS) [43]. The antioxidant capacity of extracts is commonly evaluated through DPPH and ABTS radical scavenging assays, which reflect the structure-related functional performance of recovered phenolic compounds [44].

Traditional methods for extracting bioactive compounds from fruit and vegetable waste include maceration, decoction, infusion, percolation, and Soxhlet extraction [45]. For example, Rifna et al. (2021) reported that carotenoids extracted from orange peel using Soxhlet extraction readily decomposed into terpenoid monomers. Similarly, phenolic compounds extracted using this method exhibited lower purity compared to certain novel extraction techniques [30].

In recent years, research and development in extraction technology have shifted their focus toward improving extraction efficiency, purity, and environmental sustainability. Among these, ultrasonic-assisted extraction has been widely used to isolate bioactive compounds from citrus peel. This technique transfers energy to the material matrix through mechanical waves, utilizing cavitation effects to disrupt cell wall structures, thereby promoting the release of target compounds [46].

Microwave-assisted extraction operates via volumetric dielectric heating. Under an alternating electromagnetic microwave field, polar molecules and dipolar components inside the plant matrix undergo rapid realignment and frictional rotation. This converts electromagnetic energy directly into thermal heat uniformly throughout the interior of raw-material particles, rather than relying on slow external heat conduction. Such internal volumetric heating rapidly generates local high-temperature micro-zones within citrus peel tissue, which ruptures cell structures and accelerates the diffusion of intracellular bioactive substances into the surrounding solvent. However, uneven field distribution in large-volume reactors may produce hot-spot effects, which can trigger thermal degradation of thermally-sensitive constituents such as d-limonene and polyphenols [47].

Table 1 comprehensively summarizes phenolic extraction performance of conventional maceration, UAE, MAE, and SFE under diversified parameter conditions, enabling multi-dimensional technological comparison based on phenolic yield, antioxidant activity, monomer composition, and compound stability. As illustrated in Table 1, extraction parameters exert significant influences on the recovery efficiency of phenolic compounds. Under different parameter combinations, the total phenolic content extracted from acid lime peel varies considerably, ranging from 3376.0 ± 563.0 mg GAE/100 g DW to 5440.0 mg GAE/100 g DW [48,49]. Optimised UAE conditions (48 °C, 56.71 W, 40 min) yielded the highest total phenolic content of 15,263.3 mg GAE/100 g DW together with a phenolic recovery rate of 26.52%. Under comparable experimental conditions, UAE generally achieves better performance than microwave-assisted extraction (MAE) and provides superior protection for the bioactivity of extracted compounds [50]. In comparison, conventional static maceration only achieves relatively low phenolic yields (2300.0 mg GAE/100 g DW), and parallel experimental results verify that UAE outperforms conventional maceration in phenolic recovery and antioxidant retention [40]. Supercritical fluid extraction (SFE) serves as another solvent-free alternative, obtaining moderate phenolic yields accompanied by strong DPPH radical scavenging activity [51].

**Table 1:** Total Phenolic Content Extracted by Different Green Methods.

Citrus Species	Extraction Technology	Power (W)	Temperature (°C)	Time (min)	Solvent System (*v*/*v*)	Total Phenolic Content (mg GAE/100 g DW)	Measured Properties of Extracts	Ref.
*Citrus* *reticulata*	UAE	-	37 ± 1	30	Samples: acetone (1:3)	2830.0	DPPH 48.23%; UAE > maceration	[40]
*Citrus* *reticulata*	conventional maceration	-	37 ± 1	30	Samples: acetone (1:3)	2300.0	DPPH 42.96%; UAE > maceration	[40]
*Citrus* *reticulata*	UAE	56.71	48	40	Acetone: water (80:20)	15,263.3	Hesperidin content: 6435.5 mg/100 g DW; UAE > MAE (1.77×)	[50]
*Citrus ×* *aurantium*	UAE	80	-	34.7	Acetone: water (80:20)	3376.0 ± 563.0	Total flavonoid content: 7550.0 mg/100 g DW; DPPH IC_50_ = 30.7 μg/mL; UAE > MAE	[48]
*Citrus* *sinensis*	UAE	133	59.83	45	water	5002.0	UAE > CE	[20]
*Citrus* *sinensis*	UAE	60	26	28	Water: methanol: DMSO (1:4:5)	2407.0 ± 119.0	UAE/EAE > CSE; phenolic fractions: FPs/EBPs/GBPs	[52]
*Citrus × aurantiifolia*	UAE	285	-	4	Ethanol: water (55:45)	5440.0	UAE > MAE	[49]
*Citrus* *reticulata*	SFE	-	43	120	Supercritical CO_2_	4122.0	DPPH 79.94%	[51]

Notes: UAE, ultrasound-assisted extraction; SFE, supercritical CO_2_ extraction; MAE, microwave-assisted extraction; CE, conventional extraction; CSE, conventional solvent extraction; EAE, enzyme-assisted extraction; DPPH, 2,2-diphenyl-1-picrylhydrazyl; IC_50_, half-maximal inhibitory concentration; GAE, gallic acid equivalent; DW, dry weight; DMSO, dimethyl sulfoxide.

In addition to the extraction method, the content of bioactive compounds is also affected by factors such as species, geographic origin, and cultivation practices, which directly alter the chemical composition of the fruit peel [38]. Therefore, the development of an extraction process should comprehensively consider raw material characteristics, ultrasonic power, temperature, extraction time, solvent system, and the storage stability of the product, so as to achieve efficient and stable extraction of active components. However, most existing studies have focused only on optimizing the parameters of a single technique, such as ultrasound or microwave extraction, and there is still a lack of quantitative comparison across four aspects: extraction efficiency, energy consumption, retention of active compounds, and industrial applicability. In addition, under the same extraction process, differences in raw materials can make experimental data difficult to compare across studies. As a result, current optimization conclusions are applicable only to specific experimental conditions, and their general applicability is clearly limited [53].

It should also be noted that organic solvents including methanol, acetone and dimethyl sulfoxide are widely used for extracting phenolic compounds from citrus peel. In line with green chemistry principles, strategies for industrial-scale solvent recovery require greater consideration. Distillation, vacuum evaporation and membrane separation represent feasible approaches to recycle these polar solvents, reducing raw material demand and waste generation. Nevertheless, trade-offs exist between separation efficiency, recovery energy costs and risks of residual solvent in final extracts. Establishing low-energy recovery technologies or adopting alternative greener solvents could further minimise the environmental burden of phenolic extraction processes.

### 3.2. Pectin

Pectin is a key carbohydrate polymer that constitutes plant cell walls, with its backbone mainly composed of linear galacturonic acid units connected by α-1,4 glycosidic bonds. As a heteropolysaccharide, pectin is widely used in the food, pharmaceutical, and personal care industries for its functional properties, such as gel formation, viscosity enhancement, and emulsion stabilization [54]. Its thickening capacity is influenced by various factors, including pH, temperature, ionic strength, co-solvents, molecular weight, and other intrinsic and extrinsic variables [55]. These factors collectively determine the suitability of pectin for various applications, including stabilizers, emulsifiers [56], functional foods [57], encapsulation carriers, and biodegradable packaging materials [58].

During fruit processing, pectin-rich peels are frequently discarded and considered low-value residues. As a result, recent research has increasingly focused on extracting pectin from by-products such as apple pomace [59], plantain peel [60], and dragon fruit peel [61]. Citrus peel generally exhibits a higher pectin content than common fruit and vegetable by-products such as dragon fruit, grape pomace [62], and watermelon [7], indicating its strong potential as a promising raw material for pectin extraction. A detailed comparison is provided in Table 2.

Nevertheless, extraction yield cannot independently reflect pectin quality. Critical quality criteria include the degree of esterification (DE) and galacturonic acid (GalA) content, which are listed in Table 2. Additionally, molecular weight distribution, rheological characteristics, and multiple functional attributes such as water-holding capacity (WHC), oil-holding capacity (OHC), emulsifying ability and gelling behaviour are strongly relevant to practical utilisation. These properties are closely associated with polysaccharide chain integrity and thermal stability [14,17,63].

Harsh acidic extraction conditions easily trigger chain hydrolysis, reduce average molecular weight and damage rhamnogalacturonan-I (RG-I) side chains, inevitably impairing emulsification, water retention and prebiotic activity. Comparatively, ultrasonic-assisted extraction and microwave-assisted extraction carried out under relatively mild conditions facilitate better structural preservation of pectin. Differences in these structural features ultimately determine gelling performance, thermal resistance and application potential in food and biomaterial fields. Therefore, comprehensive evaluation of pectin properties rather than merely yield is essential when screening suitable extraction technologies [14,15]. It is worth noting that although several studies have characterised DE and GalA values of citrus pectin, molecular weight distribution data are rarely reported in published literature, underscoring the urgency of establishing a more comprehensive multi-parameter evaluation system.

**Table 2:** Different methods for the extraction of citrus peel pectin.

Extraction Method	Citrus Species	Extraction Conditions	Yield (%)	DE (%)	GalA (%)	Ref.
Citric acid extraction	*Citrus reticulata*	3% acidified methanol, 70 °C, 120 min	23.1	58.38	65.47	[16]
Citric acid extraction	*Citrus maxima*	0.55% acidified solution, 150 min	24.0	-	-	[64]
MAE	*Citrus sinensis*	900 W, 160 °C, 5 min	35.76	72.17	73.48	[63]
MAE	*Citrus limetta*	600 W, 3 min	32.75	63.20	67.93	[17]
UAE	*Citrus* *reticulata*	600 W, 90 °C, 30 min	30.59	80.09	68.21	[14]
UAE	*Citrus* *limon*	70 W, 42.65 min	32.17	81.89	-	[15]

Notes: MAE, microwave-assisted extraction; UAE, ultrasound-assisted extraction; DE, degree of esterification; GalA, galacturonic acid.

Kumari et al. (2023) extracted and characterized pectin from citrus peel and evaluated its prebiotic properties [14]. The study compared three extraction methods: water extraction, citric acid extraction, and ultrasonic-assisted extraction. The results indicated that citric acid extraction not only yielded higher pectin but also better preserved the side-chain structure of the rhamnogalacturonan-I (RG-I) segment. This resulted in enhanced bacterial proliferation and improved probiotic efficacy. In addition, physicochemical characterization revealed that UAE produced pectin with the highest DE (80.09%), anhydrogalacturonic acid (AUA 73.40%), and GalA (68.21%), indicating superior purity and esterification compared to conventional methods.

Tuan et al. (2019) systematically adjusted the citric acid concentration at a fixed temperature to optimize the extraction process [64]. The results showed that increasing the citric acid concentration from 0.11% to 0.55% (which corresponded to a pH decrease from 3.4 to 2.1) boosted pectin yield from 16.3% to 24%. Liew et al. (2016) further confirmed this trend, observing an increase in pectin yield from 8.56% to 32.41% as pH decreased from 2.30 to 1.70 [65]. Similarly, another study reported that pectin yield varied between 4.47% and 39.57% as pH decreased from 2.5 to 1.5. This is primarily attributed to increased ion participation in the hydrolysis of the pectin matrix at lower pH, which promotes release. However, yields decrease when the pH falls below 1.5. Overall, citric acid is an effective solvent for extracting pectin from pomelo peel [66] (Liew et al., 2018). It should be noted, however, that these studies primarily focused on yield optimization without providing detailed characterization of DE, GalA, or molecular weight, limiting a comprehensive quality assessment.

Iñiguez-Moreno et al. (2025) investigated the microwave-assisted extraction of pectin from orange peel [63]. Under maximum power conditions, extraction time, pH, temperature, and liquid-to-solid ratio significantly affected the yield. The study confirmed that this method produces high-quality pectin with favorable yields. At the optimal conditions (160 °C, 5 min), the extracted pectin exhibited a DE of 77.27%, AUA of 67–75%, equivalent weight (EW) of 264.97 g/mol, and methoxyl content (MTC) of 4.30%, confirming its classification as high-methoxyl pectin (HMP). Sharma et al. (2023) also employed microwave-assisted extraction (microwave power: 600 W, pH: 1, duration: 3 min) to extract pectin from *Citrus limetta* peel [17]. They reported a DE of 63.20%, AUA of 67.93%, EW of 798.45 g/mol, MTC of 8.06%, WHC of 6.27 g water/g pectin, and OHC of 2.68 g oil/g pectin, further confirming the HMP nature and good functional properties of the recovered pectin. Both studies validated the effectiveness of microwave-assisted extraction, although optimal process parameters varied. This suggests that irradiation time, power, and other variables must be adjusted based on specific raw materials and equipment. Additionally, combining multiple techniques may enhance efficiency.

Sharma et al. (2023) and Spinei & Oroian (2022) further demonstrated that increasing microwave power enhances pectin yield, a phenomenon confirmed in grapefruit, sour orange, papaya, and banana peels [17,47]. This mechanism is attributed to increased microwave energy absorption by the material, which accelerates solvent heating and pectin release.

Kumari et al. (2023) explored ultrasonic-assisted extraction of citrus pectin, emphasizing that, in addition to temperature, the combined use of citric acid and ultrasound is crucial for determining yield and quality [14]. In their study, UAE yielded pectin with the highest DE (80.09%), AUA (73.40%), and GalA (68.21%), along with the highest EW (726.07 mg), indicating that ultrasound not only improves extraction efficiency but also preserves the structural integrity of the pectin molecule. Karbuz & Tugrul (2020) similarly emphasized that, in comparing lemon, orange, and kiwi peel extractions, a comprehensive consideration of all process variables—particularly the selection of acid type—is essential for achieving optimal extraction efficiency [67].

Singhal et al. (2024) employed high-power ultrasound to extract pectin from Assam lemon (*Citrus limon*) peels, yielding 32.17% [15]. The extracted pectin exhibited a DE of 81.89%, confirming its HMP classification, though this was slightly lower than the DE obtained by CE (90.29%) and MAE (84.28%) within the same study, likely due to prolonged extraction time causing partial de-esterification. Notably, DE values of citric-acid-extracted pectin vary substantially across independent publications, which is primarily controlled by citrus species, extraction pH, temperature and duration. Despite variations in specific operational parameters and yields across studies, ultrasonic technology is widely regarded as a more sustainable extraction method than traditional hot water or acid-base approaches, owing to its high safety, low energy consumption, and short processing time. This consensus has been widely accepted within the academic community, and the prospects of UAE for pectin recovery from agro-industrial residues have been comprehensively reviewed [68].

Acid extraction yields a higher pectin recovery, yet severe hydrolysis may reduce molecular weight and easily damage the RG-I side-chain structure, negatively affecting rheological behaviour, WHC, OHC and emulsifying capacity. Ultrasound- and microwave-assisted extraction operate under milder conditions and better preserve the functional properties of pectin, but they require higher equipment investment and operating energy consumption. Most existing studies focus predominantly on maximising extraction yield, while simultaneous characterisation of DE, GalA content, molecular weight, rheology, thermal stability and functional performance remains limited. Studies have only optimized individual processes separately and have not comprehensively evaluated production cost and product application requirements, making it difficult to directly guide industrial pectin production [15].

Molecular-weight distribution is another vital quality marker; strong acid hydrolysis reduces molecular weight and impairs pectin rheological behaviour. Rheological performance and thermal stability of pectin are highly sensitive to extraction intensity. Harsh acid-thermal treatments fragment polysaccharide chains and weaken thermal tolerance, while mild UAE and MAE help retain molecular structure, better thermal stability and functional indices including WHC, OHC, emulsifying and gelling properties [47].

### 3.3. Essential Oils

Citrus peel essential oil is a complex mixture primarily composed of terpenes, sesquiterpenes, and their oxygenated derivatives, including aldehydes, ketones, and alcohols. d-limonene is the predominant component, accounting for over 70% of the total content, with concentrations varying by citrus species [69,70]. These volatile aromatic oils are primarily stored in the epidermal oil glands of the peel. Their content and chemical composition are significantly influenced by cultivar, origin, fruit maturity, and extraction method [71].

Due to their distinctive aromas and bioactive properties, citrus peel essential oils have extensive applications in food flavoring, pharmaceuticals, cosmetics, and as natural antimicrobial and antioxidant agents [72]. In the global citrus processing industry, the peel is a primary byproduct and an ideal raw material for extracting these high-value compounds. In recent years, research on extracting essential oils from various citrus varieties, such as lemon and grapefruit peels, has intensified. A particular focus has been placed on developing green insecticides and food preservatives [69,73].

Extraction methods for citrus peel essential oils vary, each with distinct characteristics that shape the volatile compound profile, limonene retention, oxidative stability and biological activity of final oil products, as summarised in Table 3. Steam distillation (SD) remains the most traditional and widely applied technique, owing to its ability to avoid organic solvent residues [74]. As shown in Table 3, SD can obtain essential oil containing up to 98.86% d-limonene, yet long-duration high-temperature treatment easily alters the volatile profile, promotes limonene oxidation and generates oxygenated degradation by-products. Solvent-based methods such as SLME can achieve a considerable essential oil yield under specific experimental conditions 1.26% [75], but residual solvent may interfere with volatile composition analysis and limit food-grade utilisation. To enhance extraction efficiency and quality, a series of novel assisted extraction techniques have been developed.

**Table 3:** Extraction methods and oil yields of citrus volatile oils (yield expressed as % *w*/*w* dry weight).

Extraction Method	Citrus Species	Solid-Liquid Ratio (*v*/*v*)	Power (W)	Time (min)	Essential Oil Yield (%)	d-Limonene (%)	Ref.
SD	*Citrus × aurantium*	-	-	180	-	98.86	[74]
SLME	*Citrus ×* *limon*	1:1	1000	15	1.26	68.15	[75]
UAE	*Citrus* *limetta*	1:10	80	20	-	97.00	[76]
MAE	*Citrus sinensis*	1:1.5	300	20	1.8	-	[77]
MAE	*Citrus ×* *limon*	1:9	1000	15	1.12	72.2	[75]

Notes: SD, steam distillation; SLME, solvent-liquid microextraction; UAE, ultrasound-assisted extraction; MAE, microwave-assisted extraction.

Ultrasonic-assisted extraction has gained significant attention due to its simplicity and high efficiency. Khandare et al. (2021) found that in lemon essential oil extraction, UAE achieved a 97% extraction rate of d-limonene at 60 °C for 20 min, compared to traditional steam distillation, while better preserving heat-sensitive components [76]. Mild operating conditions benefit the preservation of heat-sensitive minor volatile constituents and improve limonene retention. Microwave-assisted steam distillation has also shown significant advantages. Golmakani and Moayyedi (2016) applied microwave-assisted hydro-distillation and solvent-free microwave extraction to extract volatile oils from lemon (*Citrus limon*) peels [75]. This approach drastically reduced extraction time from 120 min in conventional methods to just 15 min, while achieving comparable yields (approximately 1.12% *v*/*w*). Bustamante et al. (2016) further demonstrated that MAE of *Citrus sinensis* peel achieved an oil yield of 1.8% [77]. Nevertheless, MAE parameters such as microwave power strongly affect volatile fingerprint: excessive localised overheating may accelerate limonene degradation and modify the balance between monoterpenes and oxygenated volatiles.

Yield results vary across studies and conditions. Conventional steam distillation of orange peel essential oil typically yields between 0.5% and 1.0% (*v*/*w*) [71]. In contrast, microwave-assisted hydro-distillation can increase yields to approximately 1.8% (*v*/*w*) [77], offering advantages in both time efficiency and energy consumption. However, due to variations in cultivar, raw material processing, and experimental design, direct comparisons of method applicability remain challenging across studies.

The dominant constituent of citrus peel essential oil, d-limonene, exhibits considerable chemical reactivity. It readily undergoes free-radical oxidation under high-temperature extraction conditions or prolonged ambient storage, generating aldehydes, ketones and other oxidation by-products that weaken the essential oil’s antimicrobial and antioxidant performance. The adopted extraction strategy directly governs limonene retention, oxidative stability and long-term storage behaviour. Conventional steam distillation involving prolonged heating causes substantial limonene degradation and poor oxidative stability. In comparison, UAE and short-duration MAE reduce thermal exposure, facilitating higher limonene retention and better preservation of intrinsic bioactivity. Current extraction investigations predominantly aim to maximise essential oil yield, whereas process optimisation targeted at protecting limonene stability remains insufficient [78]. Accordingly, future research should not only establish baseline extraction parameters tailored to various citrus cultivars and exploit the merits of multi-process synergistic extraction, but also place greater emphasis on heat-triggered oxidative degradation of d-limonene [79]. Traditional steam distillation leaves no solvent residues, but prolonged exposure to high temperatures can cause oxidation and degradation of limonene, thereby reducing the antimicrobial and antioxidant activities of the essential oil, altering volatile profiles and weakening oxidative stability during later storage. Ultrasound- and microwave-assisted extraction can greatly shorten thermal exposure time and are more favourable for preserving active components; however, integrated equipment suitable for continuous production lines is still scarce. Most existing studies have only compared oil yield, and quantitative data on the long-term storage stability of essential oils obtained by different processes are lacking, making it difficult to establish a unified standard for process evaluation. Overall, these extraction technologies can be compared across multiple dimensions: volatile compound profile, limonene retention, oxidative stability, biological activity, storage stability and industrial applicability. SD is industrially mature and solvent-free yet risks altering volatile profiles and degrading limonene. SLME provides high yield but suffers solvent-residue concerns limiting food-grade application. UAE and short-time MAE favour better limonene retention, preservation of volatile fingerprints and bioactivity with improved oxidative stability, while their industrial scale-up is still constrained by equipment limitations [71,80].

### 3.4. A Multidimensional Comparative Analysis of Candidate Green Extraction Technologies

A comprehensive comparison of heat reflux extraction, acid extraction, ultrasound-assisted extraction, microwave-assisted extraction, and supercritical CO_2_ extraction should be made across seven dimensions: extraction efficiency, product quality, energy consumption, environmental burden, scale-up difficulty, equipment investment, and industrial maturity. In terms of extraction efficiency, UAE generally provides the highest pectin yield among the investigated extraction techniques. Singhal et al. (2024) reported a yield of 32.17% from Assam lemon peels using UAE, compared to 19.61% for MAE and 16.56% for conventional extraction under optimized conditions [15]. Similarly, Kumari et al. (2023) achieved 30.59% yield via UAE from kinnow peels, outperforming MAE (26.87%), acid-assisted extraction (AAE, 20.46%), and aqueous extraction (18.57%) [14]. For pomelo peels, Tuan et al. (2019) obtained pectin yields ranging from 11% to 24% depending on citric acid concentration (0–0.55%), while MAE at 900 W for 5 min yielded 35.76% from orange peels [63,64]. In terms of product quality, all extracted pectins exhibited DE values exceeding 50%, confirming their classification as high-methoxyl pectin (HMP). UAE produced pectin with the highest DE (80.09%) and GalA content (68.21%) in kinnow peels, while MAE yielded DE values ranging from 63.20% to 90.29% depending on raw material and conditions [14,15,17]. Iñiguez-Moreno et al. (2025) reported that MAE yielded pectin with DE of 77.27%, AUA of 67–75%, and EW of 264.97 g/mol, confirming high purity standards (AUA > 65%) [63]. Notably, however, molecular weight data remain absent from many cited studies, limiting comprehensive quality comparison.

Conventional extraction equipment is inexpensive and suitable for continuous production lines, but it has high energy consumption and causes severe loss of active compounds. For example, Kumari et al. (2023) quantified energy consumption for pectin extraction from kinnow peels: AAE consumed 2430 kJ per kg peel powder, MAE consumed 90 kJ, and UAE consumed 1 098 kJ (based on 600 W for 30 min). While MAE achieved the lowest energy input (90 kJ), UAE consumed approximately 12 times more energy than MAE despite producing a higher yield [14]. The cost of energy per kg of pectin was estimated at 5.36 INR for AAE, 0.16 INR for MAE, and 2.40 INR for UAE, with MAE being the most energy-efficient option. Regarding water and solvent consumption, conventional and acid extraction typically require longer extraction times (90–120 min) and larger solvent volumes (solid-to-liquid ratios up to 1:70), generating substantial acidic wastewater that requires neutralization and treatment before disposal. In contrast, MAE and UAE significantly reduce processing time (3–7 min for MAE, 30–45 min for UAE) and solvent usage, thereby minimizing liquid waste generation. However, complete life-cycle assessment (LCA) quantification-covering water consumption, electricity usage, wastewater treatment, and carbon footprint-remains unavailable for most systems, representing a critical gap for sustainability evaluation.

Ultrasound- and microwave-assisted extraction, regarded as relatively green processes, operate under milder conditions and provide higher recovery of target compounds, but they require expensive scale-up equipment and lack well-established solvent recovery systems. From a techno-economic perspective, while MAE and UAE offer higher extraction efficiency and better product quality, their industrial adoption is hindered by high capital investment (probe-type ultrasonicator, microwave reactor systems) and the absence of integrated solvent recovery loops, which increase overall production costs. Supercritical extraction offers the highest product purity, but the operating and maintenance costs of high-pressure equipment are substantial, creating a relatively high barrier to commercialization [18]. Additionally, most laboratory-scale parameters have not been evaluated together with the full-chain costs of raw material pretreatment (drying, grinding, sieving), solvent consumption, and product purification (ethanol precipitation), making direct industrial implementation difficult [81].

In terms of scalability, conventional heat reflux and acid extraction enjoy excellent scalability; laboratory parameters can be relatively easily translated into continuous industrial reactors. By contrast, batch probe ultrasonication and microwave reactors face prominent scale-up barriers. Uneven energy distribution in large vessels leads to non-uniform cavitation for UAE and inconsistent dielectric heating for MAE, often causing obvious efficiency decline after amplification [81]. Supercritical CO_2_ extraction is restricted by limited high-pressure vessel volume, low single-batch throughput and costly sealing components, which limits its large-scale processing of low-cost citrus by-products [82]. From a sustainability perspective, these extraction processes differ substantially in life-cycle water and electricity demand as well as solid and liquid waste output. Nevertheless, systematic full-chain LCA quantification including carbon footprint is still rarely reported in current citrus-processing by-products extraction literature [83]. For instance, conventional acid extraction generates large volumes of acidic filtrate (up to 70 mL solvent per g peel powder) that require neutralization, while ethanol precipitation-common to all methods-produces organic wastewater that demands recovery and reuse systems to reduce environmental burden [83,84]. Coupled and hybrid extraction processes (e.g., ultrasound-microwave synergy, sequential extraction of multiple bioactives) represent the main direction for industrial development [81], as a single extraction process cannot simultaneously achieve high yield, low cost, and low pollution. Future research should prioritize standardized LCA and techno-economic analysis to evaluate environmental footprint and economic viability across different scales, providing actionable guidance for industrial stakeholders.

No single extraction technology can simultaneously achieve high extraction efficiency, superior product quality, low energy input, minor environmental burden and low capital investment. Hybrid synergistic extraction routes combined with standardized LCA and techno-economic evaluation frameworks represent the most promising direction for future industrial transformation of citrus-processing by-products valorization.

Up to now, only a limited number of studies have implemented full-chain LCA for citrus-processing by-products biorefinery following ISO 14040 guidelines. Existing LCA investigations commonly identify biomass drying and solvent separation as primary environmental hotspots [85]. However, many studies simplify supply-chain variables such as seasonal raw-material storage and transportation costs. Furthermore, most assessments only evaluate a single target product (pectin alone), rarely accounting for co-product benefits within integrated cascading extraction routes. Consistent cross-study comparison is hampered by inconsistent functional units and system boundaries. Accordingly, unified evaluation protocols covering cascading multi-component extraction are urgently required to support fair sustainability assessment for industrial translation.

## 4. Applications

The diversified high-value utilization of plant by-products is a current research focus, aiming to promote the conversion of agricultural and industrial by-products into high-value applications and improve resource utilization efficiency. For example, corn husks can be used to develop biodegradable films [86], sugarcane bagasse is suitable for preparing biochar catalysts [87] (Gopinath et al., 2018), and apple and grape processing waste can yield natural antioxidants and antimicrobial compounds [8,88].

**Figure 3:**
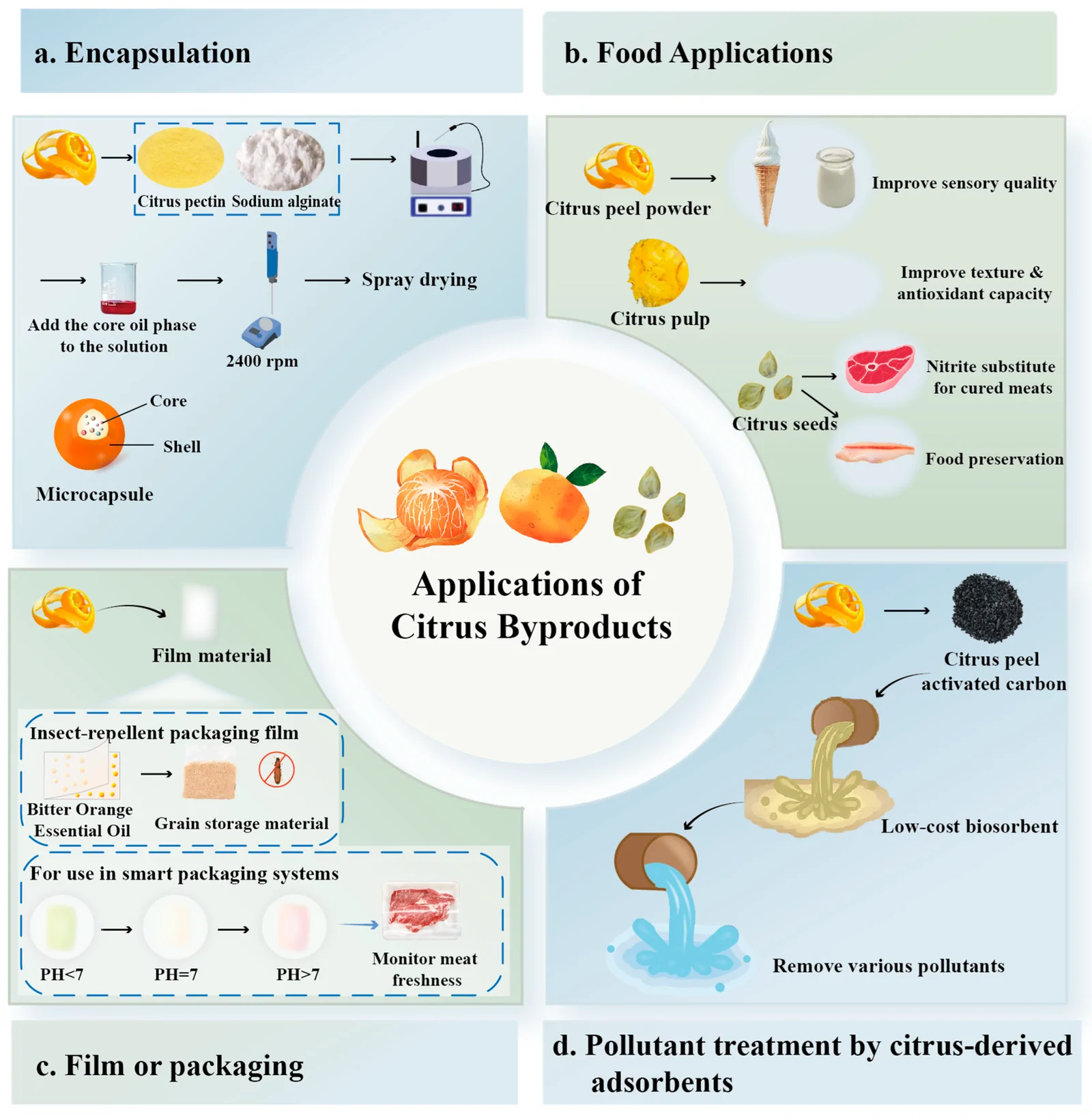
Multiple applications of citrus-processing by-products. (**a**) Microcapsule fabrication by encapsulation and spray-drying using citrus pectin; (**b**) Food-oriented applications for sensory improvement, antioxidant enhancement and food preservation; (**c**) Preparation of functional films and smart packaging for grain storage and meat-freshness monitoring; (**d**) Production of citrus-peel-derived biosorbent for pollutant removal.

Among various plant by-products, citrus peel extract exhibits exceptional functional properties. It is rich in diverse bioactive compounds with significant antioxidant capacity and contains macromolecules such as pectin and mucilage, which function as stabilizers and emulsifiers, performing essential physical roles. These characteristics offer broad application potential in the food, pharmaceutical, and cosmetic sectors (Figure 3). Furthermore, studies indicate that the addition of citrus peel powder effectively enhances the nutritional quality of food and improves physicochemical properties, such as texture, water-holding capacity, and stability.

### 4.1. Encapsulation

Encapsulation technology utilizes functional carriers to encapsulate active substances, protecting them from degradation during processing and long-term storage, thereby enhancing product stability and bioavailability [89]. Particles produced by this technology are classified by size into microparticles (>1 μm) and nanoparticles (<1 μm). Both structures consist of two fundamental components: the encapsulated active substance forms the core, while a continuous membrane or coating made of wall material surrounds it, protecting it from external influences [36]. Common preparation methods include microsponge solidification, nanoprecipitation, spray-drying, emulsion encapsulation, and emulsion solvent evaporation [90].

Encapsulant selection has expanded from traditional materials such as carbohydrates, gums, cellulose, and waxes to include emerging food processing by-products. Recent studies have demonstrated that hybrid crude palm oil [91], passion fruit peel pectin [92], and dragon fruit peel mucilage can either be directly utilized or have their carbohydrates extracted for encapsulation, offering new avenues for developing sustainable barrier materials [93]. El Basett et al. (2025) successfully encapsulated lycopene micelles using a water-in-oil emulsion system composed of citrus peel pectin combined with sodium alginate [9]. This pectin exhibited superior protective effects on lycopene during particle processing, showing higher retention rates and antioxidant efficacy compared to the conventional wall material maltodextrin. This advantage is mainly attributed to the enhanced emulsifying properties and structural stability of citrus pectin.

Similarly, Oro et al. (2023) found that when microencapsulating Brazilian Cherokee blackberry pulp extracts using various wall materials, a composite Arabic gum-pectin wall material demonstrated superior encapsulation efficacy compared to a single pectin wall material [94]. This composite system exhibited controlled-release properties for phenolic compounds during digestion, with a retention rate exceeding 51%, indicating its potential as an antioxidant and natural pigment for applications in the food, pharmaceutical, and cosmetic industries.

Further research indicates that pectin extracted from citrus peel is an excellent microencapsulation material for encapsulating active components derived from citrus, such as tangeretin [57]. Using citrus pectin/sodium alginate as the wall material to encapsulate bergamot oil at a concentration of 0.2%, the retention and encapsulation efficiencies of naringin reached 71.05% and 75.64%, respectively. This demonstrates that citrus peel inherently contains abundant bioactive compounds and serves as an ideal source of wall materials for encapsulating these compounds, achieving high-value circular utilization via the encapsulation and protection of peel-derived bioactive compounds using peel-based wall materials.

### 4.2. Food Applications

Citrus peel contains high levels of dietary fiber, polyphenolic compounds (including hesperidin and naringin), and bioactive components such as essential oils. In recent years, it has received growing attention in the food industry as a high-value functional ingredient and as a substitute for conventional raw materials. Studies show that citrus peel extracts can improve food texture, enhance antioxidant activity, replace synthetic additives, and extend shelf life.

The use of citrus peel in dairy products has shown notable benefits. Tomar and Akarca (2019) reported that adding 0.5% citrus peel essential oil to ice cream provided antibacterial and antifungal effects [95]. Sensory acceptability remained comparable to that of the control, even when fat content was reduced. Alamoudi et al. (2022) found that incorporating 5% orange peel pulp into yogurt increased lactic acid bacteria counts by 58% compared with the control [10]. The addition also improved physicochemical indicators, including fat content, thereby enhancing product stability and texture and increasing overall sensory quality.

Xie and Zhang (2023) examined the use of citrus by-products as ingredient substitutes [60]. They used a blend of dragon fruit peel extract and lemon seed essential oil as a natural antioxidant to partially replace nitrites in cured meats. The treated group showed significantly lower lipid oxidation than the nitrite control, indicating that the natural extract can reduce nitrite usage and its associated health risks.

M. Kaur et al. (2021) evaluated the effects of adding 20% orange pulp powder on pasta quality [96]. The addition improved yield and post-cooking firmness, reduced lipid oxidation, increased bioactive compound content, and produced a deeper product color. Thus, broader use of citrus by-products may support the functionalization and naturalization of food formulations without reducing consumer acceptance.

Bioactive compounds derived from citrus peel offer significant value in food preservation because of their antioxidant, coloring, and stabilizing properties. Ucak et al. (2021) used gelatin films with 2% lemon seed and sweet orange seed extracts to package sea bass fillets [97]. This treatment extended shelf life by 6 days under refrigeration at 4 ± 1 °C. Sujiwo et al. (2025) reported that adding 0.18% calamansi extract to chicken nuggets stored at 4 °C improved product quality and extended shelf life to 19 days while maintaining acceptable sensory attributes [98]. These findings support the feasibility and effectiveness of citrus extracts in practical preservation applications.

### 4.3. Films or Packaging

As the environmental impact of food packaging materials becomes more pronounced, developing biodegradable and sustainable alternatives has emerged as a key research focus. Citrus peel, a significant byproduct of the juice and food processing industries, shows promising potential in biopolymer films and functional coatings due to its high content of valuable components, including flavonoids, pectin, and volatile oils.

Flavonoids in citrus peel are naturally occurring, water-soluble polyphenols with potential as pH-responsive indicators, making them ideal for smart packaging systems. Shoja et al. (2023) incorporated 1.5% citrus flavonoid extract into a chitosan/polyvinyl alcohol composite film, significantly enhancing its mechanical properties, UV barrier performance, antioxidant capacity, and antimicrobial activity [11]. This film extended the shelf life of refrigerated sturgeon fillets and enabled real-time quality monitoring. Similarly, Guo et al. (2024) developed a pectin-soy protein isolate composite film containing citrus flavonoids [99]. This film exhibits strong antioxidant properties and high sensitivity to pH and ammonia gas, undergoing a visible color change from pale yellow to yellowish-brown during pork spoilage, thus indicating meat freshness.

Pectin, another key component of citrus peel, is highly sensitive to extraction conditions, which directly influence the final film properties. Karim et al. (2022) systematically investigated the effects of extraction temperatures (70–90 °C) and pH levels (1.0–3.0) on the chemical characteristics and film-forming ability of lemon pectin [58]. They found that extraction parameters significantly affected the mechanical properties and antioxidant activity of pectin films, but had negligible effects on water vapor barrier properties, water absorption, transparency, and thermal stability. Further research revealed that adding silica to pectin films reduced water sensitivity, enhanced tensile strength and water vapor barrier properties, and improved thermal stability.

To optimize film performance, pectin is commonly blended with other polymers or nanomaterials. Dash et al. (2019) prepared biodegradable composite films by blending lemon pectin with sweet potato starch and incorporating titanium dioxide nanoparticles [100]. As TiO_2_ concentration increased, the film exhibited enhanced tensile strength and Young’s modulus, reduced water vapor transmission and solubility, and developed a granular rough surface structure. Sadeghi et al. (2025) developed a gelatin/pectin composite film containing orange peel carbon dots and hibiscus anthocyanins [101]. This film exhibited enhanced mechanical properties and produced a significant color reaction to ammonia within 25 min, making it suitable for visual monitoring of red meat spoilage.

Volatile oils from citrus peel are widely used in active packaging. Djebbi et al. (2023) developed an insecticidal film based on a pectin matrix loaded with bitter orange essential oil [102]. This film achieved an 80.6% encapsulation rate of essential oil, demonstrating 100% lethality against adult grain moths and completely inhibiting the emergence of their offspring during storage. It also maintained wheat grain germination rates and storage quality, demonstrating its potential as a green grain protection material.

In summary, citrus peel serves as a source of pectin for biopolymer matrices, and its functional components—such as flavonoids and volatile oils—provide diverse pathways for developing active and smart packaging systems, demonstrating its value in sustainable food packaging.

In addition to functional performance, multiple safety and regulatory considerations should be addressed for broader industrial translation of citrus peel-derived materials. Raw citrus peel processing by-products may carry inherent risks including pesticide residues, surface microbial contamination, and endogenous allergenic compounds [103,104,105]. When organic solvents are adopted during phytochemical extraction, residual solvent levels require strict monitoring to meet threshold limits [105]. At present, unified technical specifications for citrus peel extracts remain incomplete; variations in cultivation origin, maturity, drying and extraction procedures lead to inconsistent composition, hindering consistent safety evaluation. Although most citrus flavonoids and pectin display low acute toxicity in preclinical tests, comprehensive long-term toxicological data, maximum permitted usage concentrations and allergenic risk assessments are still limited. Specifically, repeated-dose toxicity, genotoxicity, reproductive and developmental toxicity studies are scarce for most citrus-derived extracts [106]. Maximum permitted use concentrations for citrus peel extracts as food additives vary across jurisdictions: the FDA generally recognizes citrus pectin and certain flavonoids as GRAS (Generally Recognized as Safe) without a specified maximum limit when used in accordance with good manufacturing practice (U.S. Food and Drug Administration, n.d.), while EFSA has established specific use levels for certain citrus-derived compounds (e.g., hesperidin: up to 250 mg/day in food supplements) (EFSA *NDA* Panel, 2024) [107,108]. In China, the National Health Commission sets concentration limits for specific citrus extracts based on the intended food category [109]. These disparate regulatory frameworks, combined with the lack of harmonized toxicological reference values, create considerable barriers to cross-border commercialization. Furthermore, obvious regional regulatory differences exist: food additive and food-contact material standards administered by the European Food Safety Authority (EFSA), the U.S. Food and Drug Administration (FDA), and China’s National Health Commission adopt distinct evaluation criteria and authorized dosage limits for plant-derived bioactive ingredients, which creates barriers to cross-border commercial application. Further harmonized standardization and systematic safety assessment will facilitate the reliable application of citrus peel materials in food, encapsulation systems and biodegradable packaging.

It should also be emphasized that most promising outcomes summarized above originate from laboratory-scale investigations, and few extraction and material fabrication routes have been fully validated at pilot or industrial scale. Multiple practical bottlenecks remain unresolved for industrial translation. Citrus peel supply exhibits strong seasonal fluctuation, while fresh peel is high-moisture, perishable and costly to transport and store [5]. Energy-intensive drying steps, high capital investment for dedicated extraction equipment, low annual equipment utilization rates, requirements for solvent recovery, downstream product purification and accompanying wastewater treatment collectively raise overall operating costs. In addition, the market competitiveness of co-products obtained from integrated biorefinery chains needs further verification. Higher extraction yields at lab scale do not guarantee economic or environmental viability in industrial operation. Accordingly, claims regarding circular economy benefits and sustainability should be stated cautiously. Robust quantitative techno-economic analysis (TEA) and life cycle assessment (LCA) under real industrial boundary conditions are required to reliably quantify environmental performance and economic profitability of citrus peel biorefinery processes [110].

### 4.4. Pollutant Treatment via Transformed Citrus By-Product Adsorbents

Beyond direct extraction and utilization of intrinsic pectin, flavonoids and essential oils, citrus-processing by-products can be further upgraded via physical and chemical transformation routes. These adsorption-oriented applications are not extraction technologies per se, but are included here because the residual solid fractions obtained after pectin, polyphenol, and essential oil extraction serve as the primary feedstock for preparing activated carbon and other biosorbents. Thus, adsorption represents a downstream valorization pathway that completes the integrated biorefinery sequence. This section is kept concise to maintain the review’s focus on extraction; detailed adsorption mechanisms are not covered.

In wastewater treatment, citrus peel is commonly processed through alkaline activation, pyrolysis, or chemical modification to produce activated carbon adsorbents with high specific surface areas. These materials efficiently remove various pollutants due to their rich organic content and porous structure [111]. Recent studies show that activated carbon derived from citrus peel treated with reagents such as CO_2_, H_2_SO_4_, and FeCl_3_ achieves adsorption efficiencies exceeding 80% for organic dyes and heavy metal ions. Table 4 summarizes recent research on the utilization of citrus peel in waste resource management and water treatment.

As a low-cost, environmentally friendly biosorbent, citrus peel shows excellent performance in treating various aquatic pollutants. Biosorbent materials derived from citrus processing by-products efficiently adsorb chromium ions (Cr^3+^) in water. Maldonado et al. (2021) found that the adsorption behavior conforms to the Sips model, with a maximum adsorption capacity of 15.3 mg/g and a Cr^3+^ removal rate of 95% [112]. Additionally, Siddique et al. (2020) reported excellent adsorption performance of citrus peel-derived activated carbon for fluoride ions (F^−^) [12]. This material also demonstrates high removal efficiency for heavy metals, such as copper ions (Cu^2+^), highlighting the potential of citrus peel-based adsorbents in industrial wastewater treatment and resource recovery [113].

Citrus-based adsorbent materials exhibit excellent adsorption performance for heavy metals and dyes in single-use applications, but their adsorption capacity declines markedly after multiple adsorption–regeneration cycles. Acid washing, thermal activation, and solvent desorption each have distinct advantages and disadvantages: thermal regeneration is relatively stable but energy-intensive, whereas solvent desorption consumes less energy but may generate secondary wastewater. Existing studies have mostly focused only on the initial adsorption efficiency, and there remains a significant gap in low-cost, reusable modification processes [114].

It should be noted that safety and regulatory requirements differ significantly between citrus peel adsorbents used for water pollutant remediation and those intended for food, pharmaceutical or food-contact packaging applications, since the former are not directly exposed to humans or food matrices.

**Table 4:** Different applications of citrus peel in waste and water treatment.

Material	Process	Treatment	Main Results	Reference
Lemon Peel—Fe_3_O_4_ Nanocomposite	Hydrothermal precipitation of Fe_3_O_4_ using	Methylene blue (MB)	93% MB removal (50 mg/L, pH 8, 15 mg adsorbent) lemon peel extract.	[115]
*Citrus limetta* (peel and pulp)	Magnetic bioadsorbents prepared from peels and pulp at 500 °C.	As (III) and As (V) removal from water	As (III): 714.28 μg/g (PAC-500), 526.31 μg/g (PPAC-500); As (V): q_max_ = 2000 μg/g for both.	[116]
Grapefruit peel	One-step synthesis of GFP@Ag bio-composite using bio-derived nano-doped peel.	Toluidine blue (TO), Crystal violet (CV), Brilliant green (BG)	Degradation/biosorption capacities: 194.8 mg/g (TO), 390.6 mg/g (CV), 306 mg/g (BG); visible light, 0.005 g adsorbent, 100–200 ppm, 20–120 min.	[117]
*Citrus limetta* peel activated carbon	H_2_SO_4_ activation to highly porous activated carbon	Methylene Blue and Crystal Violet Dyes	82.77% MB and 89.87% CV removal (pH 7, 0.1 g, 120 min).	[118]
Citrus fruits wastes (orange, mandarin, rangpur lime, sweet lime)	Pyrolysis followed by H_2_O or CO_2_ activation	Cu(II) removal	BET surface area: 212.4 m^2^/g (CO_2_) and 399.4 m^2^/g (H_2_O); Cu(II) adsorption capacity: 28.2 and 27.8 mg/g, respectively.	[113]
Orange peel (*Citrus sinensis*)	Drying (45 °C, 72 h) and grinding	Cr(III) removal	Maximum adsorption capacity: 15.3 mg/g; 95% removal achieved.	[112]
*Citrus limetta* peel FeCl_3_-activated	FeCl_3_ activation and carbonization at 250 °C and 500 °C.	Fluoride removal	Langmuir capacity: 4.926 mg/g (AC-CLP250) and 9.709 mg/g (AC-CLP500); optimal: pH 6.6, 1.0 g/L, 240 min.	[12]
*Citrus × limon* peel- based La–Ce bimetallic	Conversion to magnetic nano/bio-adsorbent loaded with Ce–La nanoparticles	Phosphate removal	>98.12% phosphate removal (20 mg/L, pH 6.68, 30 °C)	[119]

Notes: MB, methylene blue; TO, toluidine blue; CV, crystal violet; q_max_, maximum adsorption capacity; BET, Brunauer-Emmett-Teller; PAC-500, pyrolyzed adsorbent carbon at 500 °C; PPAC-500, pulp-derived pyrolyzed adsorbent carbon at 500 °C.

## 5. Discussion and Future Perspectives

### 5.1. Discussion

#### 5.1.1. Raw Material Heterogeneity and Standardisation Gaps

Existing studies on citrus-processing by-products valorization share three limitations: lack of multi-dimensional benchmarks and unified evaluation frameworks, predominant laboratory-scale testing without industrial feasibility assessments, and neglect of real-world variables such as raw-material heterogeneity, safety regulations, and adsorption-regeneration cycles [18]. Chemical composition varies considerably with cultivar, origin, growing conditions, and maturity, yet few studies compare multiple genotypes under identical extraction parameters. Inconsistent reporting of extraction conditions and product quality further impedes direct cross-study comparisons.

#### 5.1.2. Green Extraction: Laboratory Advantages Versus Scale-Up Barriers

Citrus by-products are rich in flavonoids (naringin, hesperidin), d-limonene-dominant essential oils, pectin, dietary fibre, and phenolic antioxidants. Despite large-scale industrial use of citrus pulp, substantial peel residues are still discarded or underutilised.

Emerging green techniques—ultrasound-assisted (UAE) and microwave-assisted extraction (MAE)—outperform conventional methods in efficiency, kinetics, and preservation of thermally-sensitive bioactives [17,48,49]. Nevertheless, laboratory-scale feasibility does not ensure industrial viability. Most published work neglects engineering challenges, including continuous operation, solvent recycling, and process integration, creating a distinct gap between bench-scale trials and pilot/industrial production.

#### 5.1.3. Application Prospects and Commercialisation Hurdles

Citrus extracts show broad potential: as natural antioxidants/antimicrobials and dietary-fibre ingredients for functional foods [96,98]; in biomaterials, as activated carbon, carbon dots, and nanofibres for electrochemical sensing, intelligent packaging, and biomedical carriers; and as feedstocks for pharmaceuticals and cosmetics. However, most applications remain laboratory-based, lacking product development and safety data for market entry [99,101,102].

Commercialisation is constrained by variable raw-material composition, unstandardised pretreatment, high capital costs for advanced equipment, seasonal supply, perishability, drying expenses, and reliance on cascading co-product value and solvent recovery for profitability. Sustainability claims therefore require quantitative life-cycle assessment (LCA) and techno-economic analysis (TEA) under realistic industrial conditions [5,81].

#### 5.1.4. Towards an Integrated Analytical Framework

To bridge the identified gaps, this review proposes an integrated framework encompassing standardised pretreatment, coupled green extraction, purification, safety evaluation, and co-production of multiple high-value products. This differentiates our work from existing reviews that focus primarily on pairwise comparisons of extraction techniques.

Crucially, the lack of harmonised reporting standards has rendered the majority of published yield data effectively non-comparable. While the literature widely advocates UAE and MAE as superior, closer examination reveals that reported enhancement ratios for identical raw materials vary substantially across studies, attributable solely to differences in solvent-to-solid ratio definitions and power density calculations. This variability fundamentally undermines the validity of meta-analytical conclusions drawn thus far. Therefore, the integrated framework proposed herein is not merely a procedural checklist; it represents a necessary shift in research perspective—moving the field from yield-centric pairwise comparisons to a systems-level assessment grounded in standardised baselines and multi-criteria decision analysis. The central argument is that translating laboratory research into industrial biorefinery demands not only technological advances but also harmonised characterisation protocols, aligned LCA-TEA methodologies, and value-chain collaboration. Operationally, this calls for a minimum reporting framework that mandates explicit documentation of solvent-to-solid ratios, power density (W/g), and particle-size distributions as essential prerequisites for any meaningful cross-study comparison. Based on the above critiques, the following roadmap is not arbitrarily assembled but derived deductively from each identified bottleneck: raw-material heterogeneity dictates standardisation as the prerequisite; engineering gaps mandate pilot validation before any economic projection; and LCA/TEA inconsistencies require methodological harmonisation as the precondition for credible sustainability claims.

### 5.2. Future Perspectives

Future research should address the above bottlenecks through a three-phase roadmap. Short-term priorities: (a) establish raw-material standardisation protocols for diverse cultivars and origins; (b) advance mild green extraction, including deep eutectic solvents (DES), particularly natural deep eutectic solvents (NaDES) [120], enzyme-assisted extraction [121], and supercritical CO_2_ extraction —particularly for biological approaches not fully covered in this review [51]; (c) develop low-cost, low-footprint workflows. Medium-term objectives: (a) optimise adsorbent regeneration and solvent recycling; (b) refine safety evaluation via standardised cell-based assays, animal studies, and clinical trials [1]; (c) conduct pilot-scale trials under continuous operation; (d) integrate LCA (ISO 14040) and TEA into process optimisation to quantify environmental and economic performance [83]. Long-term goals: construct a complete citrus-peel biorefinery chain validated by comprehensive TEA and LCA [122], while developing high-value peel-based materials and composite bioactive mixtures exploiting their antioxidant, anti-inflammatory, and antibacterial properties.

### 5.3. Limitations of the Present Review

This narrative review synthesises laboratory-scale literature on physical-assisted green extraction of citrus-peel bioactives; several limitations should be acknowledged.

First, high inter-study heterogeneity (cultivar, origin, maturity, pretreatment, extraction parameters) prevents quantitative cross-comparison. Second, available datasets are predominantly small-scale; pilot- and industrial-scale evidence is scarce, and bench-optimised parameters often fail under continuous production due to uneven energy distribution, equipment constraints, and drying/transportation/solvent costs. Third, existing LCA reports use inconsistent functional units and system boundaries, and most studies prioritise extraction yield over comprehensive characterisation (molecular weight, rheology, thermo-oxidative stability, end-use performance). Fourth, this review focuses mainly on physical-assisted extraction; biological methods (enzyme-assisted, DES and NaDES) are only briefly noted in Future Perspectives without in-depth analysis. Finally, grey literature, conference abstracts, and non-English publications were excluded, potentially introducing selection bias.

## 6. Conclusions

This review critically examines the disconnection between laboratory-scale extraction research and industrial biorefinery of citrus-processing by-products. The central thesis is that the current literature’s predominant focus on pairwise technology comparisons has reached diminishing returns; without standardised raw-material baselines, harmonised LCA-TEA frameworks, and validated pilot-scale data, further bench-scale optimisation yields progressively limited industrial relevance.

Three substantive conclusions are drawn. First, UAE and MAE are technologically superior to conventional methods in efficiency and bioactivity preservation, yet this advantage is economically contingent—it converts into industrial viability only when coupled with solvent-recovery systems and cascading valorisation of multiple co-products. Second, the widely reported extraction yields are artefacts of specific lab configurations, not intrinsic material properties; cross-study generalisation is therefore statistically unsound unless reporting standards are unified. Third, sustainability claims absent TEA and ISO 14040-aligned LCA constitute an evidence gap that critically undermines their translational value.

To close the lab-to-industry gap, this review advances an integrated analytical framework—spanning standardised pretreatment, coupled green extraction, purification, safety evaluation, and multi-product co-production—as a unifying operational paradigm. The accompanying short-, medium-, and long-term roadmap provides actionable benchmarks for researchers and industrial stakeholders. We contend that the future of citrus-peel biorefinery lies not in discovering yet another extraction condition but in validating integrated process chains under real-world economic and environmental constraints.

## Data Availability

All data generated or analyzed during this study are included in this published article.

## References

[1] Maqbool Z., Khalid W., Atiq H.T., Koraqi H., Javaid Z., Alhag S.K., Al-Shuraym L.A., Bader D.M.D., Almarzuq M., Afifi M. (2023). Citrus Waste as Source of Bioactive Compounds: Extraction and Utilization in Health and Food Industry. Molecules.

[2] FAO (2024). The State of Food and Agriculture: Moving Forward on Food Loss and Waste Reduction. https://www.fao.org/publications/sofa/en/.

[3] Durmus N., Gulsunoglu-Konuskan Z., Kilic-Akyilmaz M. (2024). Recovery, Bioactivity, and Utilization of Bioactive Phenolic Compounds in Citrus Peel. Food Sci. Nutr..

[4] Sharma P., Vishvakarma R., Gautam K., Vimal A., Kumar Gaur V., Farooqui A., Varjani S., Younis K. (2022). Valorization of citrus peel waste for the sustainable production of value-added products. Bioresour. Technol..

[5] Zema D.A., Calabrò P.S., Folino A., Tamburino V., Zappia G., Zimbone S.M. (2018). Valorisation of citrus processing waste: A review. Waste Manag..

[6] Kumari P., Gaur S.S., Tiwari R.K. (2023). Banana and its by-products: A comprehensive review on its nutritional composition and pharmacological benefits. EFood.

[7] Brahmi F., Chennit B., Batrouni H., Benallaoua K., Madani K., Boulekbache-Makhlouf L. (2023). Valorization of apricot, melon, and watermelon by-products by extracting vegetable oils from their seeds and formulating margarine. OCL.

[8] Gracey P.R., Tako E. (2025). Apple and grape pomace: Emerging upcycled functional ingredients in processed meat products, designed to increase polyphenol and fiber contents. Sustain. Food Technol..

[9] El Basett H., El Fihry N., Ruyssen T., De Meulenaer B., Hajjaj H. (2025). Microencapsulation of lycopene from tomato peels industrial by-product in a citrus pectin mixture matrix: Evidences on physicochemical and stability profile. Eur. Food Res. Technol..

[10] Alamoudi S.A., Saad A.M., Alsubhi N.H., Alrefaei G.I., Al-Quwaie D.A., Binothman N., Aljadani M., Alharbi M., Alanazi H., Babalghith A.O. (2022). Upgrading the physiochemical and sensory quality of yogurt by incorporating polyphenol-enriched citrus pomaces with antioxidant, antimicrobial, and antitumor activities. Front. Nutr..

[11] Shoja B., Mirzaei A.M., Atef-Alavi B., Rokni A.N. (2023). The effect of chitosan coating enriched with orange peel (*Citrus sinensis*) waste extract on extending preservation and chemical and functional properties of refrigerated beluga sturgeon (Huso huso) fillet. Arch. Razi Inst..

[12] Siddique A., Nayak A.K., Singh J. (2020). Synthesis of FeCl3-activated carbon derived from waste *Citrus limetta* peels for removal of fluoride: An eco-friendly approach for the treatment of groundwater and bio-waste collectively. Groundw. Sustain. Dev..

[13] Wang Z., Yang B., Chen X., Huang P., Chen K., Ma Y., Agarry I.E., Kan J. (2022). Optimization and comparison of nonconventional extraction techniques for soluble phenolic compounds from brocade orange (*Citrus sinensis*) peels. J. Food Sci..

[14] Kumari M., Singh S., Chauhan A.K. (2023). A Comparative Study of the Extraction of Pectin from Kinnow (*Citrus reticulata*) Peel Using Different Techniques. Food Bioprocess Technol..

[15] Singhal S., Deka S.C., Koidis A., Hulle N.R.S. (2024). Standardization of extraction of pectin from Assam lemon (*Citrus limon* Burm f.) peels using novel technologies and quality characterization. Biomass Convers. Biorefinery.

[16] Bala M., Shivani, Awasthi A., Kalsi B.S., Goswami D., Mridula D., Vishwakarma R.K., Kumar A. (2024). An innovative approach to biomass utilization through concurrent hesperidin and pectin extraction from immature dropped kinnow (*Citrus reticulata*) fruits. J. Food Meas. Charact..

[17] Sharma P., Osama K., Varjani S., Farooqui A., Younis K. (2023). Microwave-assisted valorization and characterization of *Citrus limetta* peel waste into pectin as a perspective food additive. J. Food Sci. Technol..

[18] Manakas P., Balafoutis A.T., Kottaridi C., Vlysidis A. (2024). Sustainability assessment of orange peel waste valorization pathways from juice industries. Biomass Convers. Biorefinery.

[19] (2006). Environmental Management—Life Cycle Assessment—Principles and Framework.

[20] Boukroufa M., Boutekedjiret C., Petigny L., Rakotomanomana N., Chemat F. (2015). Bio-refinery of orange peels waste: A new concept based on integrated green and solvent free extraction processes using ultrasound and microwave techniques to obtain essential oil, polyphenols and pectin. Ultrason. Sonochem..

[21] Job J.T., Visakh N.U., Pathrose B., Alfarhan A., Rajagopal R., Thayyullathil J., Thejass P., Ramesh V., Narayanankutty A. (2023). Chemical composition and biological activities of the essential oil from *Citrus reticulata* Blanco peels collected from Agrowastes. Chem. Biodivers..

[22] Bursać Kovačević D., Gajdoš Kljusurić J., Putnik P., Vukušić T., Herceg Z., Dragović-Uzelac V. (2016). Stability of polyphenols in chokeberry juice treated with gas phase plasma. Food Chem..

[23] Qian Y., Gao Z., Wang C., Ma J., Li G., Fu F., Guo J., Shan Y. (2021). Effects of Different Treatment Methods of Dried Citrus Peel (Chenpi) on Intestinal Microflora and Short-Chain Fatty Acids in Healthy Mice. Front. Nutr..

[24] Huang C.-H., Hsiao S.-Y., Lin Y.-H., Tsai G.-J. (2022). Effects of Fermented Citrus Peel on Ameliorating Obesity in Rats Fed with High-Fat Diet. Molecules.

[25] Shehata M.G., Awad T.S., Asker D., El Sohaimy S.A., Abd El- Aziz N.M., Youssef M.M. (2021). Antioxidant and antimicrobial activities and UPLC-ESI-MS/MS polyphenolic profile of sweet orange peel extracts. Curr. Res. Food Sci..

[26] Lv X., Zhao S., Ning Z., Zeng H., Shu Y., Tao O., Xiao C., Lu C., Liu Y. (2015). Citrus fruits as a treasure trove of active natural metabolites that potentially provide benefits for human health. Chem. Cent. J..

[27] Suri S., Singh A., Nema P.K. (2022). Current applications of citrus fruit processing waste: A scientific outlook. Appl. Food Res..

[28] Tanujaya M.B., Ahmadi F., Beyatli A., Imran A., Suleria H.A.R. (2025). Antioxidant capacity and polyphenol compounds characterization through LC-ESI-QTOF-MS/MS in citrus peels from different varieties. J. Food Biochem..

[29] Zaky A.A., Akram M.U., Rybak K., Witrowa-Rajchert D., Nowacka M. (2024). Bioactive compounds from plants and by-products: Novel extraction methods, applications, and limitations. AIMS Mol. Sci..

[30] Rifna E.J., Misra N.N., Dwivedi M. (2021). Recent advances in extraction technologies for recovery of bioactive compounds derived from fruit and vegetable waste peels: A review. Crit. Rev. Food Sci. Nutr..

[31] Putnik P., Bursać Kovačević D., Režek Jambrak A., Barba F., Cravotto G., Binello A., Lorenzo J., Shpigelman A. (2017). Innovative “Green” and Novel Strategies for the Extraction of Bioactive Added Value Compounds from Citrus Wastes—A Review. Molecules.

[32] He Z., Yang C., Yuan Y., He W., Wang H., Li H. (2023). Basic constituents, bioactive compounds and health-promoting benefits of wine skin pomace: A comprehensive review. Crit. Rev. Food Sci. Nutr..

[33] Ikram A., Rasheed A., Ahmad Khan A., Khan R., Ahmad M., Bashir R., Hassan Mohamed M. (2024). Exploring the health benefits and utility of carrots and carrot pomace: A systematic review. Int. J. Food Prop..

[34] Andrade R.C., Ferreira Ribeiro C.D., Caetano V.C.D.S., Fernandes S.S., Otero D.M. (2024). Trends in dragon fruit peel compound extraction and technological applications. Trends Food Sci. Technol..

[35] Peng Q., Zhang Y., Zhu M., Bao F., Deng J., Li W. (2022). Polymethoxyflavones from citrus peel: Advances in extraction methods, biological properties, and potential applications. Crit. Rev. Food Sci. Nutr..

[36] Ozkan G., Ceyhan T., Çatalkaya G., Rajan L., Ullah H., Daglia M., Capanoglu E. (2024). Encapsulated phenolic compounds: Clinical efficacy of a novel delivery method. Phytochem. Rev..

[37] Keawmanee N., Ma G., Zhang L., Kato M. (2023). Regulation of Chlorophyll and Carotenoid Metabolism in Citrus Fruit During Maturation and Regreening. Rev. Agric. Sci..

[38] Zhu C., Zhou X., Long C., Du Y., Li J., Yue J., Pan S. (2020). Variations of Flavonoid Composition and Antioxidant Properties among Different Cultivars, Fruit Tissues and Developmental Stages of Citrus Fruits. Chem. Biodivers..

[39] Kim D.S., Lim S.B. (2020). Extraction of flavanones from immature *Citrus unshiu* pomace: Process optimization and antioxidant evaluation. Sci. Rep..

[40] Saini A., Panesar P.S., Bera M.B. (2019). Comparative Study on the Extraction and Quantification of Polyphenols from Citrus Peels Using Maceration and Ultrasonic Technique. Curr. Res. Nutr. Food Sci. J..

[41] Alara O.R., Abdurahman N.H., Ukaegbu C.I. (2021). Extraction of phenolic compounds: A review. Curr. Res. Food Sci..

[42] Munteanu I.G., Apetrei C. (2021). Analytical methods used in determining antioxidant activity: A review. Int. J. Mol. Sci..

[43] Brits M., Naessens T., Theunis M., Taktak O., Allouche N., Pieters L., Foubert K. (2021). Identification and quantification of polymethoxylated flavonoids in different citrus species using UPLC-QTOF-MS/MS and HPLC-DAD. Planta Medica.

[44] Tan J.B.L., Lim Y.Y. (2015). Critical analysis of current methods for assessing the in vitro antioxidant and antibacterial activity of plant extracts. Food Chem..

[45] Parra-Pacheco B., Cruz-Moreno B.A., Aguirre-Becerra H., García-Trejo J.F., Feregrino-Pérez A.A. (2024). Bioactive Compounds from Organic Waste. Molecules.

[46] Khedmat L., Izadi A., Mofid V., Mojtahedi S.Y. (2020). Recent advances in extracting pectin by single and combined ultrasound techniques: A review of techno-functional and bioactive health-promoting aspects. Carbohydr. Polym..

[47] Spinei M., Oroian M. (2022). Microwave-assisted extraction of pectin from grape pomace. Sci. Rep..

[48] Abdallah H.B., Abbassi A., Trabelsi A., Krichen Y., Chekir-Ghedira L., Ghedira K. (2023). Optimization of ultrasound-assisted extraction of polyphenols and flavonoids from *Citrus aurantium* L. var. amara Engl. fruit peel using response surface methodology. Biomass Convers. Biorefinery.

[49] Rodsamran P., Sothornvit R. (2019). Extraction of phenolic compounds from lime peel waste using ultrasonic-assisted and microwave-assisted extractions. Food Biosci..

[50] Nipornram S., Tochampa W., Rattanatraiwong P., Singanusong R. (2018). Optimization of low power ultrasound-assisted extraction of phenolic compounds from mandarin (*Citrus reticulata* Blanco cv. Sainampueng) peel. Food Chem..

[51] Rajput S., Kaur S., Panesar P.S., Thakur A. (2022). Supercritical fluid extraction of essential oils from *Citrus reticulata* peels: Optimization and characterization studies. Biomass Convers. Biorefinery.

[52] Wang Z., Mei X., Chen X., Rao S., Ju T., Li J., Yang Z. (2023). Extraction and recovery of bioactive soluble phenolic compounds from brocade orange (*Citrus sinensis*) peels: Effect of different extraction methods thereon. Lwt.

[53] Duggal M., Singh D.P., Singh S., Khubber S., Garg M., Krishania M. (2024). Microwave-assisted acid extraction of high-methoxyl kinnow (*Citrus reticulata*) peels pectin: Process, techno-functionality, characterization and life cycle assessment. Food Chem. Mol. Sci..

[54] Wang W., Ma X., Xu Y., Cao Y., Jiang Z., Ding T., Ye X., Liu D. (2015). Ultrasound-assisted heating extraction of pectin from grapefruit peel: Optimization and comparison with the conventional method. Food Chem..

[55] Setyajati F.D.E., Prasetyo V.K., Husin A.S., Ratri M.C., Junedi S., Sanjayadi S., Setiawati A. (2023). Comparative physicochemical properties of isolated pectin from various tropical fruit peel wastes. Trop. J. Nat. Product. Res..

[56] Yang X., Liu X., Zhao S., Huo M., Tian G., Sang Y. (2024). Pectin from steam explosion-treated citrus peel exhibits good emulsion properties and bioavailability-promoting effect in vitro of nobiletin. Int. J. Biol. Macromol..

[57] Sun X., Cameron R.G., Manthey J.A., Hunter W.B., Bai J. (2020). Microencapsulation of Tangeretin in a Citrus Pectin Mixture Matrix. Foods.

[58] Karim R., Nahar K., Zohora F.T., Islam M.M., Bhuiyan R.H., Jahan M.S., Shaikh M.A.A. (2022). Pectin from lemon and mango peel: Extraction, characterisation and application in biodegradable film. Carbohydr. Polym. Technol. Appl..

[59] Wu B., Gao K., Guo Y., Ma Y., Qiu C., Song C., Ma H. (2023). Research progress on extraction of active components from apple processing waste. Crit. Rev. Food Sci. Nutr..

[60] Xie J., Zhang Y., Klomklao S., Simpson B.K. (2023). Pectin from plantain peels: Green recovery for transformation into reinforced packaging films. Waste Manag..

[61] Chua B.L., Tang S.F., Ali A., Chow Y.H. (2020). Optimisation of pectin production from dragon fruit peels waste: Drying, extraction and characterisation studies. SN Appl. Sci..

[62] Megías-Pérez R., Ferreira-Lazarte A., Villamiel M. (2023). Valorization of Grape Pomace as a Renewable Source of Techno-Functional and Antioxidant Pectins. Antioxidants.

[63] Iñiguez-Moreno M., Pizaña-Aranda J.J.P., Ramírez-Gamboa D., Ramírez-Herrera C.A., Araújo R.G., Parra-Saldívar R., Melchor-Martínez E.M. (2025). Multitarget optimization of citrus pectin extraction using microwave-assisted technique and citric acid. Biomass Convers. Biorefinery.

[64] Tuan N.T., Dang L.N., Huong B.T.C., Danh L.T. (2019). One step extraction of essential oils and pectin from pomelo (*Citrus grandis*) peels. Chem. Eng. Process. Process Intensif..

[65] Liew S.Q., Ngoh G.C., Yusoff R., Teoh W.H. (2016). Sequential ultrasound-microwave assisted acid extraction (UMAE) of pectin from pomelo peels. Int. J. Biol. Macromol..

[66] Liew S.Q., Ngoh G.C., Yusoff R., Teoh W.H. (2018). Acid and Deep Eutectic Solvent (DES) extraction of pectin from pomelo (*Citrus grandis* (L.) Osbeck) peels. Biocatal. Agric. Biotechnol..

[67] Karbuz P., Tugrul N. (2021). Microwave and ultrasound assisted extraction of pectin from various fruits peel. J. Food Sci. Technol..

[68] Cano-Gonzalez C.N., Herrera-Estrada M.D.L.L., Contreras-Esquivel J.C., Soriano-Melgar L.D.A.A., Aguirre-Loredo R.Y. (2024). Valorization of Agro-Industrial Wastes for Ultrasound-Assisted Extraction of Pectin: A Review. Sustainable Agriculture and Global Environmental Health.

[69] Meryem S., Mohamed D., Nour-eddine C., Faouzi E. (2023). Chemical composition, antibacterial and antioxidant properties of three Moroccan citrus peel essential oils. Sci. Afr..

[70] Zheng W., Wu F., Luo X., Bian C., Zhang Y., Yin F., Liu H., Fu Y. (2022). Separation of Citrus Essential Oil and Hesperidin from Citrus Peel Through Combined Mixed-Solvent and Multi-Phase Salting Out Extraction. J. Essent. Oil Bear. Plants.

[71] Gavahian M., Chu Y.H., Mousavi Khaneghah A. (2018). Recent advances in orange oil extraction: An opportunity for the valorisation of orange peel waste a review. Int. J. Food Sci. Technol..

[72] Grover S., Aggarwal P., Kumar A., Kaur S., Yadav R., Babbar N. (2024). Utilizing citrus peel waste: A review of essential oil extraction, characterization, and food-industry potential. Biomass Convers. Biorefinery.

[73] Silwanyana Y., Mazwi V., Miya G., Oriola A.O., Hosu Y.S., Oyedeji A.O., Oyedeji O.O., Kuria S.K. (2025). Effectiveness of citrus essential oils as a biopesticide against stored food product pests: A review. J. Essent. Oil Bear. Plants.

[74] Ashmawy N.S., Nilofar N., Zengin G., Eldahshan O.A. (2024). Metabolic profiling and enzyme inhibitory activity of the essential oil of *Citrus aurantium* fruit peel. BMC Complement. Med. Ther..

[75] Golmakani M.-T., Moayyedi M. (2016). Comparison of microwave-assisted hydrodistillation and solvent-less microwave extraction of essential oil from dry and fresh *Citrus limon* (Eureka variety) peel. J. Essent. Oil Res..

[76] Khandare R.D., Tomke P.D., Rathod V.K. (2021). Kinetic modeling and process intensification of ultrasound-assisted extraction of d-limonene using citrus industry waste. Chem. Eng. Process. Process Intensif..

[77] Bustamante J., van Stempvoort S., García-Gallarreta M., Houghton J.A., Briers H.K., Budarin V.L., Matharu A.S., Clark J.H. (2016). Microwave assisted hydro-distillation of essential oils from wet citrus peel waste. J. Clean. Prod..

[78] Belem B.R., Carapeto G.V., Issa M.G., Ferraz H.G. (2023). Microemulsions: An Encapsulation Strategy to Increase the Thermal Stability of d-limonene. Pharmaceutics.

[79] Bernart M.W. (2015). Ultra-high performance liquid chromatography (UHPLC) method for the determination of limonene in sweet orange (*Citrus sinensis*) oil: Implications for limonene stability. J. AOAC Int..

[80] Siddiqui S.A., Pahmeyer M.J., Assadpour E., Jafari S.M. (2022). Extraction and purification of d-limonene from orange peel wastes: Recent advances. Ind. Crops Prod..

[81] Núñez-Pérez J., Burbano-García J.L., Espín-Valladares R., Lara-Fiallos M.V., DelaVega-Quintero J.C., Cevallos-Vallejos M., Pais-Chanfrau J.-M. (2026). Cascade valorisation of lemon processing residues (Part II): Integrated biorefinery design, circular economy, and techno-economic feasibility. Foods.

[82] Santos V.D.M., Calado V.M.A., Bojorge N. (2022). Oil separation process of *Citrus sinensis* orange: Simulation and techno-economic evaluation. Biofuels Bioprod. Biorefining.

[83] Midolo G., Cutuli G., Porto S.M.C., Ottolina G., Paini J., Valenti F. (2024). LCA analysis for assessing environmental sustainability of new biobased chemicals by valorising citrus waste. Sci. Rep..

[84] Vasconcellos M.S., Saavedra Y.M.B. (2026). Systematic literature review of orange waste management from the environmental life cycle perspective. Biomass Bioenergy.

[85] Spada E., Calabrò P.S., De Luca A.I., Gulisano G., Iofrida N., Mauriello F., Falcone G. (2026). Circular bioenergy from citrus waste: Life cycle evaluation in the Mediterranean context. Biomass Bioenergy.

[86] Chawla P., Sridhar K., Kumar A., Sarangi P.K., Bains A., Sharma M. (2023). Production of nanocellulose from corn husk for the development of antimicrobial biodegradable packaging film. Int. J. Biol. Macromol..

[87] Gopinath A., Bahurudeen A., Appari S., Nanthagopalan P. (2018). A circular framework for the valorisation of sugar industry wastes: Review on the industrial symbiosis between sugar, construction and energy industries. J. Clean. Prod..

[88] Sette P., Fernandez A., Soria J., Rodriguez R., Salvatori D., Mazza G. (2020). Integral valorization of fruit waste from wine and cider industries. J. Clean. Prod..

[89] Yan C., Kim S.-R. (2024). Microencapsulation for Pharmaceutical Applications: A Review. ACS Appl. Bio Mater..

[90] Xu Y., Yan X., Zheng H., Li J., Wu X., Xu J., Zhen Z., Du C. (2024). The application of encapsulation technology in the food Industry: Classifications, recent Advances, and perspectives. Food Chem. X.

[91] Assunção L.S., Oliveira de Souza C., Shahidi F., Santos Oliveira T., Assis D.D.J., Pereira Santos L.F., Nunes I.L., Machado B.A.S., Ferreira Ribeiro C.D. (2024). Optimization and Characterization of Interspecific Hybrid Crude Palm Oil Unaué HIE OxG Nanoparticles with Vegetable By-Products as Encapsulants. Foods.

[92] Li X., Lin Y., Huang Y., Li X., An F., Song H., Huang Q. (2024). Preparation and characterization of zein–caseinate–pectin complex nanoparticles for encapsulation of curcumin: Pectin extracted by high-speed shearing from passion fruit (*Passiflora edulis f. flavicarpa*) peel. J. Sci. Food Agric..

[93] Otálora M.C., Wilches-Torres A., Gómez Castaño J.A. (2023). Microencapsulation of Betaxanthin Pigments from Pitahaya (*Hylocereus megalanthus*) By-Products: Characterization, Food Application, Stability, and In Vitro Gastrointestinal Digestion. Foods.

[94] Oro C.E.D., Paroul N., Mignoni M.L., Zabot G.L., Backes G.T., Dallago R.M., Tres M.V. (2023). Microencapsulation of Brazilian Cherokee blackberry extract by freeze-drying using maltodextrin, gum Arabic, and pectin as carrier materials. Food Sci. Technol. Int..

[95] Tomar O., Akarca G. (2019). Effects of ice cream produced with lemon, mandarin, and orange peel essential oils on some physicochemical, microbiological and sensorial properties. Kocatepe Vet. J..

[96] Kaur M., Dhaliwal M., Kaur H., Singh M., Punia Bangar S., Kumar M., Pandiselvam R. (2021). Preparation of antioxidant-rich tricolor pasta using microwave processed orange pomace and cucumber peel powder: A study on nutraceutical, textural, color, and sensory attributes. J. Texture Stud..

[97] Ucak I., Abuibaid A.K.M., Aldawoud T.M.S., Galanakis C.M., Montesano D. (2021). Antioxidant and antimicrobial effects of gelatin films incorporated with citrus seed extract on the shelf life of sea bass (*Dicentrarchus labrax*) fillets. J. Food Process. Preserv..

[98] Sujiwo J., Jung Y., Lee S., Kim D., Lee H.-J., Oh S., Choo H.-J., Jang A. (2025). The Effectiveness of Calamansi (*Citrus microcarpa*) Extract in Enhancing the Shelf Life and Quality of Dakgalbi Made from Domestic Korean Chicken. Food Sci. Anim. Resour..

[99] Guo H., Bai J., Jin X., Liu H., Wu D., Gan R., Gao H. (2024). Innovative edible films for food preservation: Combining pectin and flavonoids from citrus peels with soy protein isolates. Lwt.

[100] Dash K.K., Ali N.A., Das D., Mohanta D. (2019). Thorough evaluation of sweet potato starch and lemon-waste pectin based-edible films with nano-titania inclusions for food packaging applications. Int. J. Biol. Macromol..

[101] Sadeghi S.M., Firoozjah R.A., Bahramian B., Jafari N., Majnouni S., Hadavifar S., Ehsani A., Khezerlou A., Tavassoli M. (2025). Orange peel–derived carbon dots and *H. sabdariffa* L. anthocyanin-integrated gelatin and pectin pH-responsive films for detection of meat freshness. Int. J. Biol. Macromol..

[102] Djebbi T., Soltani A., Chargui H., Sadraoui-Ajmi I., Teka N., Majdoub H., Jemâa J.M.B. (2023). Formulation of pectin-*Citrus aurantium* essential oil-based packaging film and assessment of insecticidal performances against *Rhyzopertha dominica* on stored wheat. J. Stored Prod. Res..

[103] Karaağaçlı H., Balkan T., Kızılarslan M., Kara K. (2025). Pesticide residues in citrus fruits from Türkiye: Assessment of distribution, matrix effects, and health risks in lemon, mandarin, and orange. J. Food Compos. Anal..

[104] Sehair N., Ahmed I., Khalid N. (2025). Isolation and identification of microbial communities from *Citrus limetta* waste. Food Saf. Health.

[105] Zamarro Parra M.S., Martínez-Gomaríz M., Hernández A., Alcover J., Dobski I., Rodríguez D., Palacios R., Carbonell A. (2025). Orange allergy beyond LTP: IgE recognition of germin-like proteins in citrus fruits. Curr. Issues Mol. Biol..

[106] Molehin O.R., Ajayi O.O., Adefegha S.A., Ogunro A.C., Fakayode A.E. (2026). Nutraceutical Potential and Toxicological Concerns of Citrus Fruit Phytoconstituents: A Review. KolaDaisi Univ. J. Appl. Sci..

[107] U.S. Food and Drug Administration 21 CFR § 184.1588 Pectins. Electronic Code of Federal Regulations. https://www.ecfr.gov/current/title-21/chapter-I/subchapter-B/part-184/subpart-B/section-184.1588.

[108] EFSA Panel on Nutrition, Novel Foods and Food Allergens (NDA) (2024). Safety of glucosyl hesperidin as a novel food. EFSA J..

[109] (2016). National Food Safety Standard—Food Additives—Naringin (Naringin Extract).

[110] Marchette I.R., Cavallari R.B.C., Rothier F.d.C., Bojorge N.I.B.R., Alhadeff E.M., Young A.F., dos Santos B.F. (2025). Economic assessment and process design to valorize citrus juice industry waste. Biofuels Bioprod. Biorefining.

[111] Ehrampoush M.H., Miria M., Salmani M.H., Mahvi A.H. (2015). Cadmium removal from aqueous solution by green synthesis iron oxide nanoparticles with tangerine peel extract. J. Environ. Health Sci. Eng..

[112] Maldonado J.A.H., Aguilera C.E.C., Hernández M.M.S., Arias A.N.A., Soto R.H. (2021). Evaluation of orange peel (*Citrus sinensis*) as a source of bioactive components and its use as a bioadsorbent. Desalin. Water Treat..

[113] da Silva M.D., da Boit Martinello K., Knani S., Lütke S.F., Machado L.M., Manera C., Perondi D., Godinho M., Collazzo G.C., Silva L.F. (2022). Pyrolysis of citrus wastes for the simultaneous production of adsorbents for Cu (II), H2, and d-limonene. Waste Manag..

[114] Nie J., Feng D., Shang J., Nasen B., Jiang T., Liu Y., Hou S. (2023). Green composite aerogel based on citrus peel/chitosan/bentonite for sustainable removal Cu(II) from water matrices. Sci. Rep..

[115] Nasser A., Kareem S.L. (2024). Removal of Congo red from aqueous solution using lemon peel-Fe_3_O_4_ nanocomposite adsorbent. Biomass Convers. Biorefinery.

[116] Verma L., Siddique M.A., Singh J., Bharagava R.N. (2019). As (III) and As (V) removal by using iron impregnated biosorbents derived from waste biomass of *Citrus limmeta* (peel and pulp) from the aqueous solution and ground water. J. Environ. Manag..

[117] Akl M.A., Elawady D.M., Mostafa A.G., El-Gharkawy E.R. (2025). Biogenic nano-silver doped grapefruit peels biocomposite for biosorptive photocatalytic degradation of organic pollutants. Sci. Rep..

[118] Rani S., Chaudhary S. (2022). Adsorption of methylene blue and crystal violet dye from waste water using *Citrus limetta* peel as an adsorbent. Mater. Today Proc..

[119] Akram M., Bano Z., Bhutto S.U.A., Pan J., Majeed M.K., Li L., Xia M., Wang F. (2025). Sustainable Synthesis of Ce-La Loaded onto *Citrus limon* for Phosphate Removal: Performance and Regeneration Studies. Colloids Surf. B Biointerfaces.

[120] Gomez-Urios C., Siroli L., Grassi S., Patrignani F., Blesa J., Lanciotti R., Frígola A., Iametti S., Esteve M.J., Di Nunzio M. (2025). Sustainable valorization of citrus by-products: Natural deep eutectic solvents for bioactive extraction and biological applications of *Citrus sinensis* peel. Eur. Food Res. Technol..

[121] Barbosa P.D.P.M., Ruviaro A.R., Macedo G.A. (2020). Conditions of enzyme-assisted extraction to increase the recovery of flavanone aglycones from pectin waste. J. Food Sci. Technol..

[122] Olmedo-Galarza V., Pinto-Mosquera N., Pineda-Flores H., Manosalvas-Quiroz L. (2025). Citrus Waste Valorization: Unconventional Pathways for Sustainable Biomaterials and Bioactive Products. Sustainability.

